# Integrative AI-assisted modeling suggests CPPF binding at a composite *α*/*β*-tubulin interface pocket dominated by *β*-tubulin contacts

**DOI:** 10.1371/journal.pcbi.1014804

**Published:** 2026-09-18

**Authors:** Jixin Yang, Lisha Liang, Dengchao Zhu, Xuzhe Yin, Yizhuo Feng, Shaojun Tang, Muzi Li

**Affiliations:** 1 Data Science and Analytics Thrust, Information Hub, The Hong Kong University of Science and Technology (Guangzhou), Guangzhou, China; 2 Bioscience and Biomedical Engineering Thrust, Systems Hub, The Hong Kong University of Science and Technology (Guangzhou), Guangzhou, China; 3 Department of Biostatistics, Virginia Commonwealth University, Richmond, Virginia, United States of America; 4 Massey Comprehensive Cancer Center, Virginia Commonwealth University, Richmond, Virginia, United States of America; 5 Shenzhen Key Laboratory of Synthetic Genomics, Guangdong Provincial Key Laboratory of Synthetic Genomics, State Key Laboratory of Quantitative Synthetic Biology, Shenzhen Institute of Synthetic Biology, Shenzhen Institutes of Advanced Technology, Chinese Academy of Sciences, Shenzhen, China; 6 Faculty of Synthetic Biology and Institute of Emerging Agricultural Technology, Shenzhen University of Advanced Technology, Shenzhen, China; Shiraz University, IRAN, ISLAMIC REPUBLIC OF

## Abstract

Microtubules are dynamic cytoskeletal polymers assembled from α/β-tubulin heterodimers. Microtubules are validated targets for novel therapeutic drugs, yet therapeutic efficacy targeting them is often compromised by multidrug resistance (MDR). 5-(3-chlorophenyl)-N-(3-pyridinyl)-2-furamide (CPPF) is a novel microtubule-targeting anticancer agent which was found to disrupt microtubule growth in cells and inhibit tubulin polymerization in vitro. CPPF could suppress the growth of multidrug-resistant cell lines and demonstrate anti-tumor efficacy in animal models. However, the fundamental mechanism of how CPPF disrupts microtubule assembly remains unclear. To investigate this, we performed structure prediction using Protenix, RoseTTAFold All-Atom (RFAA) and Umol, as well as molecular dynamics (MD) simulations, using the human α/β-tubulin heterodimer (PDB ID: 5IJ0) for analyzing the interaction between CPPF and tubulin. Our results showed that CPPF binds at the α/β interface with dominant contributions from β-tubulin residues, particularly VAL236 and LEU253. In isolated-monomer comparisons, β-tubulin exhibited more favorable binding energetics and deeper, broader free-energy minima than α-tubulin. Supplementary simulations on the alternative β-tubulin conformational state (PDB: 6E7B; straight microtubule-lattice) indicated that CPPF binding is preserved across the two major β-tubulin conformations, with comparable MM-PBSA binding free energies. Taken together, these findings establish a plausible binding mode for CPPF at the α/β-tubulin interface, providing a structural hypothesis for understanding its anticancer potential against multidrug-resistant cancers. Experimental validation through binding assays or crystallography is warranted to further substantiate these computational insights.

## Author summary

Microtubules are essential for cell division and intracellular transport, which makes tubulin a major target for anticancer drugs. However, many tumors become resistant to commonly used microtubule-targeting agents, creating a need for new compounds with distinct binding mechanisms. CPPF is a recently reported microtubule inhibitor that remains active in multidrug-resistant cancer cell lines, but how it interacts with tubulin at the molecular level is still unclear. Here, we used an integrated computational framework that combines AI-based protein–ligand structure prediction (Protenix, RoseTTAFold All-Atom (RFAA), and Umol), binding-pocket analysis, residue-level interaction profiling, and all-atom molecular dynamics simulations to characterize CPPF–tubulin recognition. Across multiple models, CPPF showed more consistent binding poses and recurrent contact residues on β-tubulin than on α-tubulin, with key hotspots including VAL236 and LEU253. Simulations further suggested that the CPPF–β-tubulin complex is dynamically more stable and occupies deeper free-energy minima than the α-tubulin complex, with an ensemble-averaged MM-PBSA binding free energy indicating a substantially favorable interaction. Complementary simulations on an alternative β-tubulin conformational state (the microtubule-lattice-related straight form) indicated that CPPF binding is preserved across both major β-tubulin conformations, suggesting the pocket is accessible at multiple stages of microtubule dynamics. Together, our results propose a plausible binding mode for CPPF at a composite α/β interface pocket with dominant contributions from β-tubulin residues, providing structural hypotheses to guide future biochemical validation and the rational optimization of tubulin inhibitors that may help overcome drug resistance.

## Introduction

Microtubules are dynamic cytoskeletal polymers playing fundamental roles in critical cellular processes such as intracellular transport, mitotic spindle formation, and directional cell migration [1–3]. Microtubules are assembled through GTP (guanosine triphosphate)-dependent polymerization of α-/β-tubulin dimers, which are the fundamental building blocks of microtubules [4]. Different α- and β-tubulin isoforms can confer unique biochemical properties and drug sensitivities, contributing to differential cellular responses to microtubule-targeting agents [5,6]. Some of these differences predominate in certain cancer cells and some are associated with the development of drug resistance [7]. For example, β3-tubulin (TUBB3) expression is up-regulated in several epithelial tumors [8] and, in lung cancer, has been associated with resistance to anti-programmed cell death protein 1 (PD-1) immunotherapy [7]. Disrupting these resistance-related processes therefore represents a promising therapeutic strategy. Microtubules are key targets in this context, with drugs such as taxanes, vinca alkaloids, and colchicine derivatives exerting anticancer effects by modulating microtubule dynamics [9]. Among the arsenal of anticancer agents, paclitaxel, marketed as Taxol, is a cornerstone drug that exerts cytotoxic effects by targeting microtubules, thereby blocking cancer cell division [10]. Paclitaxel binds to β-tubulin near the lateral interface between protofilaments [11–13], stabilizing this flexible region. Importantly, this lateral interface is inherently dynamic; experimentally, microtubules can exhibit a range of 12–15 protofilaments [14,15] and tubulin dimers can switch between curved (unpolymerized) and straight (lattice-incorporated) conformations depending on their nucleotide state and binding environment [16–19].

5-(3-chlorophenyl)-N-(3-pyridinyl)-2-furamide (CPPF) was first identified in 2020 as a potent microtubule-targeting anticancer compound [20]. It was demonstrated that CPPF could inhibit microtubule growth in vitro and in vivo [20]. CPPF induced apoptotic cell death in various cancer cell lines from different origins (lung, breast, etc.), and remained effective against multidrug resistance (MDR) cancer cell lines, which are resistant to known microtubule-targeting drugs such as paclitaxel and colchicine [20]. Structural docking suggests the potential binding site of CPPF overlaps with the colchicine binding site at the α/β-tubulin interface, a region critical for microtubule depolymerization due to its hydrophobic nature [20]. This site’s potential to facilitate CPPF binding, combined with its efficacy in MDR cells, highlights its therapeutic promise. Furthermore, CPPF demonstrated antitumor efficacy by suppressing tumor growth in different animal models, and displayed low toxicity in initial tests [20]. However, the mechanism by which CPPF disrupts microtubule dynamics was unclear.

To understand the principle of how the novel microtubule inhibitor, CPPF, interacts with α/β-tubulin, we selected the high-resolution cryo-EM structure of the human α/β-tubulin heterodimer (PDB ID: 5IJ0) as a biologically relevant template [21]. We specifically selected 5IJ0 because it captures a soluble α/β-tubulin heterodimer conformation relevant to microtubule-destabilizing ligands, which can prevent the conformational transitions required for productive lattice incorporation. This dimer was strategically selected due to the distinct expression profiles and functional roles of TUBA1B (α1B-tubulin) and TUBB3 (β3-tubulin). TUBA1B is frequently used in structural and biochemical studies of microtubules [6,11], while TUBB3 is predominantly expressed in neurons and has been linked to cancer progression, chemoresistance, and altered drug response [6,8,22].

Due to the lack of high-resolution experimental structures for CPPF–tubulin complexes, we employed a structure-based computational strategy. Deep learning methods for biomolecular structure prediction, such as RoseTTAFold All-Atom (RFAA) [23], enable modeling of assemblies containing proteins and small molecules from amino acid sequence and ligand chemical structure. RFAA extends the RoseTTAFold framework to predict three-dimensional protein–ligand complex conformations [23]. Protenix is a deep learning framework for protein–ligand docking and binding site prediction developed by ByteDance. Protenix is specifically designed to predict how a small molecule binds to a protein. It uses a geometric deep learning approach to model the complex interactions and steric constraints at a protein’s binding pocket, directly outputting the likely 3D binding pose of the ligand [24]. In our study, Protenix and RFAA provide strong capabilities for specifying ligand properties, selecting binding pockets, and customizing residue-level constraints, making them suitable for structure-guided docking tasks.

Building on this, we elucidated the atomic-level binding behavior of CPPF through a structure-based computational approach integrating ligand docking, residue-level interaction analysis, and molecular dynamics (MD) simulations. We first identified potential binding pockets on the α1B/TUBB3 heterodimer using ProteinsPlus (https://proteins.plus) [25], followed by protein–ligand complex modeling using Protenix, Umol [26], and RFAA, covering both dimeric and monomeric forms. The predicted binding poses were benchmarked using the known microtubule inhibitor nocodazole for validation.

To further dissect molecular interactions, key interacting residues were identified through spatial contact analysis and the Protein–Ligand Interaction Profiler (PLIP) [27], while Proteins, Interfaces, Structures and Assemblies (PDBePISA) [28] was employed to characterize interface energetics. Finally, we performed all-atom MD simulations in Groningen Machine for Chemical Simulations (GROMACS) [29] to assess the structural stability of the predicted complexes and constructed free energy landscapes (FELs) to probe conformational dynamics. This multi-platform framework enables the structural characterization of CPPF–tubulin interactions, bridging the gap between functional assays and atomic-resolution insight.

The contributions of this work are threefold: (1) **methodological**, we demonstrate a fit-for-purpose AI-assisted protein–ligand modeling strategy for large, flexible systems such as the tubulin heterodimer, benchmarked against experimental crystal structures with sub-1.0 Å agreement for known tubulin–ligand poses; (2) **analytical**, we introduce a reproducible, end-to-end Nextflow [30] pipeline integrating pocket analysis, multi-platform pose generation, replicate MD simulations, free-energy landscape construction, and MM-PBSA energetics; with all analysis scripts and trajectories publicly deposited, this workflow provides an open-access template that can be readily applied to other drug–target systems; and (3) **scientific**, we propose a structural model in which CPPF occupies a composite α/β interface pocket with dominant contributions from β-tubulin residues, spatially distinct from the canonical colchicine-bound pose. Additionally, supplementary MD simulations on the β-GTP straight lattice conformation (PDB: 6E7B) suggest this pocket remains accessible across the two major β-tubulin states, offering a potential rationale for CPPF’s activity in colchicine-resistant cancer cell lines. Modeling the binding behavior of CPPF offers a structural hypothesis for how it may disrupt microtubule dynamics, helping guide future experimental validation.

## Materials and methods

### Code and data availability

The computational workflow, scripts, and project documentation are available at github.com/jasperyeoh/integrative-ai-assisted-modeling-of-cppf-tubulin-interactions.

The computational workflow is additionally implemented as a reproducible Nextflow (v26.04.1) [30] pipeline available under workflows/ in the same repository, with conda environment specifications and full documentation for end-to-end reproduction.

All-atom MD production trajectories are available on Hugging Face: huggingface.co/datasets/jasperyeoh2/MD-trajectories-CPPF-tubulin-heterodimer-and-monomers.

### Ligand physicochemical analysis and tubulin structure preparation

**Fig 1:**
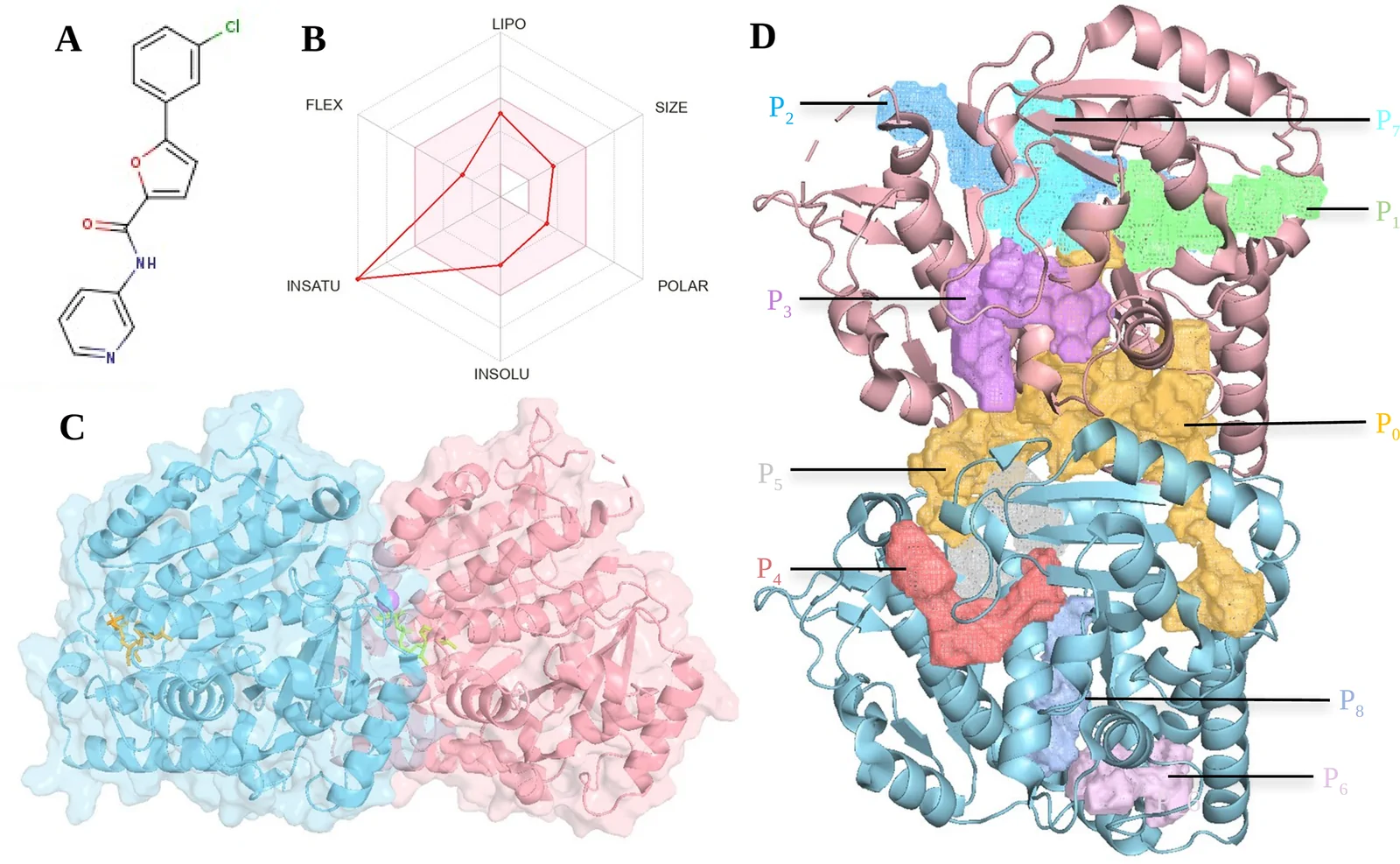
Structural and physicochemical overview of CPPF and the α/β-tubulin heterodimer. **(A)** 2D structure of the small-molecule compound CPPF. **(B)** Physicochemical profile of CPPF represented as a radar plot, including properties such as polarity, lipophilicity, solubility, and molecular size, computed via SwissADME. **(C)** Overall tertiary structure of the α1B/β3 tubulin heterodimer (PDB ID: 5IJ0), visualized in PyMOL. The α-subunit is shown in pink and the β-subunit in blue. Magnesium ions (purple spheres), GTP (lemon sticks), and GDP (orange sticks) are included as cofactors in the native structure. **(D)** Predicted ligand-binding pockets identified by ProteinsPlus. Nine surface-accessible pockets (P_0_ to P_8_) are rendered in distinct colours and mapped onto the tubulin heterodimer. Pockets with Drug Scores > 0.7 are considered druggable. P_2_ exhibited the highest Drug Score (0.9) and is located near the α/β interface, whereas P_0_, despite its large volume, is likely occluded in the dimerized state. (See S1 Table for detailed quantitative pocket parameters.).

The cryo-EM structure of the α1B/β3-tubulin heterodimer (PDB ID: 5IJ0) was retrieved from the Protein Data Bank as the structural template for all modeling tasks [21]. Structural visualization was performed in PyMOL (https://pymol.org) [31], where the α-subunit was rendered in pink and the β-subunit in blue. Magnesium ions (Mg^2+^) were shown as purple spheres, and the bound nucleotide ligands, GTP and GDP were depicted in lemon and orange stick representations, respectively. The chemical structure of CPPF [20] was constructed in SMILES format using SwissADME [32], and its physicochemical properties, including lipophilicity, polarity, solubility, and flexibility, were evaluated using radar-based descriptors. Predicted toxicity and off-target activity profiles of CPPF were obtained using ProTox 3.0 [33] and are summarized in S1 Fig. The 2D structure and physicochemical profile of CPPF, along with the tertiary structure of the α/β-tubulin heterodimer, are shown in Fig 1A–1C.

### Binding pocket analysis

To identify potential ligand-binding sites for CPPF on the tubulin heterodimer, we employed ProteinsPlus (https://proteins.plus), a structure-based platform that evaluates surface geometry and physicochemical parameters of binding pockets [25]. The software computes properties such as volume, depth, surface curvature, and hydrogen bond donor-to-acceptor ratio, which jointly contribute to the estimation of druggability scores. These structural features, particularly large, enclosed cavities with favorable hydrogen bonding potential, are typically associated with strong ligand-binding affinity. The resulting pockets provided spatial constraints for subsequent docking and validation steps.

### Protein-ligand complex structure prediction

We employed multiple structure prediction platforms, including Protenix [24], Umol [26], and RFAA [23], to model the binding conformations of CPPF with the α1B/TUBB3 heterodimer, as well as the individual α- and β-subunits. For each platform, the SMILES representation of CPPF and the corresponding amino acid sequences of the protein targets were provided as inputs. Protenix and RFAA automatically generated multiple sequence alignments and three-dimensional structural models for protein–ligand complexes.

Nocodazole, a well-characterized microtubule-destabilizing agent with an experimentally determined crystal structure binding mode [34], was used as a positive control to benchmark the modeling pipeline. The predictive accuracy of Protenix was further benchmarked by comparing Protenix-predicted nocodazole–β-tubulin complexes against the experimental crystal structure of nocodazole-bound β-tubulin (PDB: 5CA1), shown in S2 Fig. The predicted poses showed strong structural agreement with the experimental binding mode, supporting the platform’s reliability for modeling CPPF–tubulin interactions. In standard protein–ligand docking and AI-based modeling workflows, crystallographic waters, ions, and non-target cofactors are routinely simplified or omitted during pose prediction, as their explicit inclusion can vary across platforms; the structural validity of this approach is supported by the nocodazole benchmark described above. While Protenix and RFAA produced reproducible pose geometries across α-, β-, and α/β-subunit models, predictions from SwissDock [35] and Chai-1 [36] were notably less stable; SwissDock in particular yielded high root-mean-square deviation (RMSD) values relative to crystallographic references. All output structures were aligned to their respective PDB references (5IJ0 for the α/β-tubulin dimer, TUBA1B for α-tubulin, and TUBB3 for β-tubulin) using PyMOL for visual inspection and subsequent structural analysis (S3 Fig).

### Key residue identification

Residue-level contact analysis was performed across all predicted CPPF and tubulin complexes using a 4 Å heavy-atom distance threshold to define ligand–protein interactions. Biopython [37] was used to identify contact residues, and PyMOL was employed for visual inspection and polar interaction annotation. Complexes derived from Protenix and RFAA, covering both monomeric and dimeric forms of α1B- and β3-tubulin were included in the ensemble. In addition to distance-based contact identification, we further analyzed representative protein–ligand complexes using the Protein–Ligand Interaction Profiler (PLIP) to detect biochemically meaningful interactions such as hydrogen bonds, π–π stacking, and salt bridges. This analysis allowed for the annotation of key interacting residues based not only on spatial proximity but also on chemical complementarity. Example PyMOL commands for optional hydrophobicity-colored cavity surfaces are available under scripts/ in the accompanying GitHub repository.

### Interface analysis

To evaluate the physical and energetic properties of the CPPF–tubulin binding interface, we used the PDBePISA [28] server to analyze protein–ligand complexes generated by RFAA. Unlike docking methods that infer binding poses, PDBePISA quantifies interactions based on existing structural models. The analysis reports key energetic metrics such as interface area, number of contacting atoms and residues, solvation energy, hydrogen bonding, and salt bridge formation, which together contribute to the Gibbs free energy of dissociation (Δ G). Models with negative Δ G values are considered stable and likely to form persistent complexes in solution.

### Molecular dynamics simulation and free energy analysis

All-atom molecular dynamics simulations were performed using GROMACS 2024.5 [29] to assess the conformational stability of CPPF-bound tubulin systems. For the α/β heterodimer, production MD was initiated from Protenix pose 1 (Table 1) after aligning the predicted protein–ligand assembly to PDB 5IJ0. The crystallographic nucleotide cofactors (GTP at the α-site, GDP at the β-site) and the Mg^2+^ ion present in 5IJ0 were not retained in the production MD topology. This protocol is justified by three considerations: (i) the CPPF pose predicted in this study localizes near the β-tubulin N-site at the α/β interface, while the GTP/GDP cofactors occupy the E-site on the opposite face of β-tubulin (~15–20 Å away), making direct steric or electrostatic ligand–nucleotide coupling negligible; (ii) experimental affinity measurements at the overlapping colchicine site indicate that ligand binding to the tubulin dimer is not measurably modulated by the β-nucleotide state, suggesting the same insensitivity for CPPF; and (iii) the indirect cofactor effect on backbone conformation is encoded upstream by the Protenix prediction, which was performed with all cofactors explicitly present. Monomer simulations used Protenix-predicted CPPF-bound α- or β-tubulin complexes aligned to their respective PDB references (TUBA1B or TUBB3). Proteins were parameterized with the AMBER99SB-ILDN force field, and the CPPF ligand was parameterized using GAFF2 with RESP2(0.5) atomic charges derived from a B3LYP/6-31G(d) optimization (Multiwfn + ACPYPE). Each protein–ligand complex was solvated in a cubic box filled with SPC216 water molecules under periodic boundary conditions, and neutralized with sodium counterions. Energy minimization was conducted using the steepest descent algorithm, followed by equilibration under NVT and NPT ensembles at 300 K. Production simulations were carried out for 200 ns for monomer systems (three replicates each for α- and β-tubulin) and 400 ns for the α/β heterodimer (three independent replicates), using a 2 fs timestep, particle mesh Ewald (PME) for long-range electrostatics, and LINCS to constrain bond lengths.

**Table 1:** Structural alignment metrics (pTM, ipTM, and RMSD) for predicted CPPF–tubulin complexes. Protenix-generated poses (pose 0–4) for CPPF in complex with α/β-tubulin dimer, α-tubulin monomer, and β-tubulin monomer were structurally aligned to their respective reference structures from the Protein Data Bank (PDB ID: 5IJ0 for the α/β-tubulin dimer; TUBA1B for α-tubulin; TUBB3 for β-tubulin). The predicted TM-score (pTM; a predicted template-modeling score reflecting global fold confidence) and inter-chain predicted TM-score (ipTM; reflecting interface confidence for multimeric complexes) were extracted from the output of AlphaFold-compatible models. Backbone root-mean-square deviation (RMSD, in Å) was computed between predicted and reference protein structures using PyMOL. Mean, minimum, and maximum values are summarized for each structural context to assess the convergence and reliability of predicted binding poses.

Predicted poses		α/β-Tub dimer			α-Tub monomer			β-Tub monomer		
		pTM	ipTM	RMSD	pTM	ipTM	RMSD	pTM	ipTM	RMSD
**RFAA**		—	—	—	—	—	0.696	—	—	0.671
**Protenix**	**pose 0**	0.9681	0.9677	1.950	0.9777	0.9894	0.878	0.9741	0.9885	0.812
	**pose 1**	0.9673	0.9669	1.318	0.9748	0.9865	1.001	0.9744	0.9865	0.841
	**pose 2**	0.9657	0.9633	1.881	0.9722	0.9852	0.872	0.9708	0.9814	0.591
	**pose 3**	0.9634	0.9616	1.813	0.9682	0.9791	0.967	0.9682	0.9815	0.589
	**pose 4**	0.9628	0.9608	1.869	0.9700	0.9776	1.028	0.9606	0.9692	0.578
**Mean**		0.9655	0.9641	1.766	0.9726	0.9835	0.949	0.9696	0.9814	0.682
**Min**		0.9628	0.9608	1.318	0.9682	0.9776	0.872	0.9606	0.9692	0.578
**Max**		0.9681	0.9677	1.950	0.9777	0.9894	1.028	0.9744	0.9885	0.841

Post-simulation analyses included the calculation of RMSD for protein backbone atoms and ligand heavy atoms (least-squares fit on backbone), minimum protein–ligand distance (mindist), and radius of gyration (Rg). Trajectories were first processed under periodic boundary conditions using a two-step procedure (trjconv -pbc nojump followed by trjconv -pbc cluster -center for dimers or -pbc mol -ur compact -center for monomers) to avoid discontinuities that can introduce RMSD/Rg artefacts. Hydrogen bond analysis between CPPF and the protein was performed using gmx hbond-legacy due to compatibility issues of the newer gmx hbond implementation with the ligand typing in these systems.

To quantify whether CPPF remained near the predicted β-tubulin pocket in monomer simulations, we computed the minimum distance between CPPF and the canonical pocket residues VAL236, LEU253, and ALA314 during the final 50 ns window (150–200 ns). For each residue, an atom group corresponding to the residue was generated and the per-frame minimum distance to CPPF was computed using gmx mindist; distances were summarized as the mean over frames in the window.

To further explore the conformational landscape sampled during simulation, two-dimensional free energy landscapes were constructed using backbone RMSD and radius of gyration (Rg) as reaction coordinates. The joint probability distribution *P*(*x*, *y*) was converted into a free energy surface using Boltzmann inversion:


$$\begin{array}{c} \Delta G(x,y)=-k_{B}T\ln P(x,y) \end{array}$$

where $k_{B}$ is the Boltzmann constant, *T* is the temperature (300 K), and *P*(*x*, *y*) is the normalized occupancy. FELs were constructed without biasing potentials. RMSD and Rg were calculated from backbone atoms. All calculations were implemented using in-house Python scripts and GROMACS trajectory tools. For the main FEL comparison, the full 200 ns trajectories from all three replicates were concatenated within each class prior to SHAM to reduce sampling noise, and α and β FELs were plotted using shared axis limits and a shared free-energy colour scale (kcal/mol; capped at 5 kcal/mol) to enable direct visual comparison.

### Supplementary 6E7B Simulations (GTP-bound β-tubulin conformational state)

To test whether CPPF retains stable binding in the alternative β-tubulin conformational state captured by PDB **6E7B** (β-GMPCPP, straight microtubule-lattice conformation), we performed an independent set of MD simulations using a protocol designed to mirror the primary 5IJ0 simulations. The CPPF–tubulin complex starting structure was generated by running Protenix with the full 6E7B cofactor complement explicitly present in the input (one GTP at the α-site, one GMPCPP/G2P at the β-site, two Mg^2+^ ions, and CPPF), so that the predicted pose reflects the cofactor-conditioned pocket geometry of the lattice conformation. All five Protenix samples passed quality thresholds (pLDDT > 94; ipTM > 0.93); the top-ranked sample (ranking score 0.9478) was selected and aligned to the 6E7B template via Kabsch superposition on common Cα atoms, yielding a backbone RMSD of 1.82 Å relative to the experimental structure. The predicted 6E7B complex and its structural overlay with the 5IJ0-predicted complex are shown in S4 Fig.

The aligned protein–CPPF complex was then prepared for production MD using the identical toolchain as 5IJ0: AMBER99SB-ILDN for the protein, GAFF2/RESP2 for CPPF (re-using the topology generated for 5IJ0, as the ligand is identical), TIP3P water in a rhombic-dodecahedral box (1.0 nm minimum distance to box edge), neutralizing Na^+^ ions, and a target physiological ionic strength of 0.15 M. As in the 5IJ0 protocol, the bound cofactors were not retained in the production topology (see rationale above). The system contained approximately 168,000 atoms in total. Energy minimization, NVT equilibration (100 ps, 300 K), and NPT equilibration (100 ps, 300 K, 1 bar) used the same MDP parameter files as 5IJ0. Three independent replicates of 200 ns each were performed. Replicates 2 and 3 were initialized from independent NVT/NPT equilibration runs (different velocity seeds) starting from the common minimized structure, ensuring trajectory independence while preserving the equilibrated geometry.

The same analysis pipeline applied to the 5IJ0 trajectories (backbone RMSD, ligand RMSD, radius of gyration, minimum CPPF–protein distance, hydrogen-bond counts, 2D free energy landscapes via Boltzmann inversion of the (RMSD, Rg) joint distribution, and MM-PBSA-GB binding free energy using gmx_MMPBSA with the same GB-OBC2 settings as the primary 5IJ0 simulations (igb = 5, intdiel = 1.0, extdiel = 78.5, ff99SB+GAFF, ~1 ns sampling over the last 50 ns)) was applied to the 6E7B trajectories, with shared axis limits and shared free-energy colour scales used to enable direct cross-system visual comparison.

## Results

### Identification and druggability analysis of binding pockets

**Fig 2:**
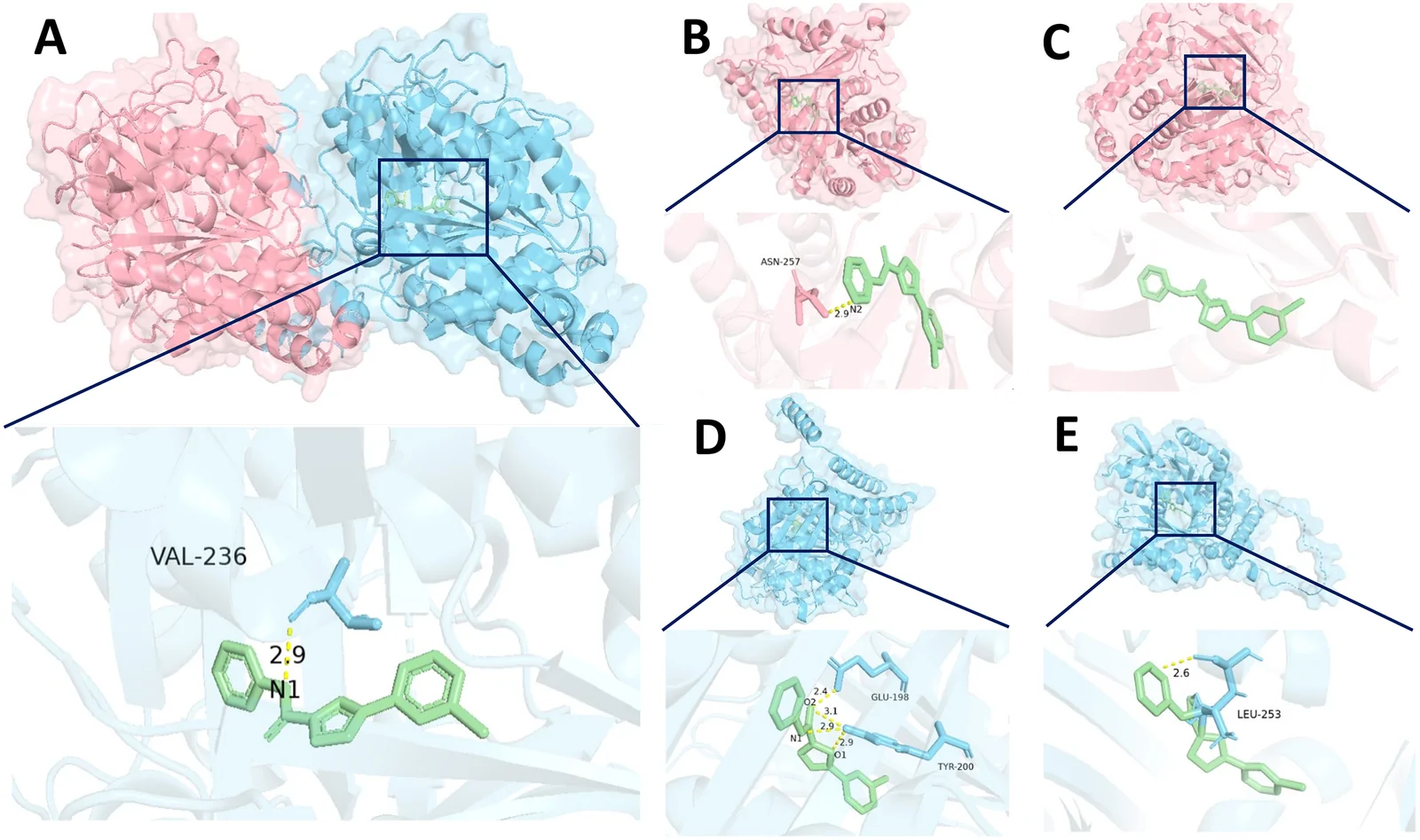
Predicted binding poses of CPPF with the α/β-tubulin dimer, α-tubulin monomer, and β-tubulin monomer from Protenix and RFAA. The predicted binding orientation between CPPF and the α/β-tubulin dimer was simulated by Protenix **(A)**. The predicted binding orientation between CPPF and α-tubulin was predicted by Protenix (B) and RFAA **(C)**. The predicted binding orientation between CPPF and β-tubulin was predicted by Protenix (D) and RFAA **(E)**. Zoom-in views below each overview show semi-transparent surfaces with representative interaction distances. Pocket localization and druggability screening are summarized in Figs 1D and 3, respectively.

To identify potential ligand-binding sites on the tubulin heterodimer, we employed ProteinsPlus (https://proteins.plus), which evaluates surface topography and chemical properties to compute pocket-specific druggability scores. A total of 29 surface pockets were detected on the α/β-tubulin dimer (PDB ID: 5IJ0), with quantitative metrics including volume, surface area, depth, polarity, and hydrophobicity for each pocket (S1 Table). To prioritize biologically relevant pockets, we applied a filtering threshold of Drug Score > 0.7, a commonly used cutoff in the DoGSiteScorer druggability-scoring scheme [38] implemented within ProteinsPlus to highlight potentially druggable pockets for downstream inspection. This yielded nine candidate pockets (P_0_–P_8_). Among these, P_2_ exhibited the highest Drug Score (0.9), with a moderate volume (559.2 Å^3^) and a favorable depth (36.79 Å), indicating strong potential to accommodate small-molecule ligands. P_1_ and P_7_ also displayed high scores (0.83 and 0.84, respectively) and were located on accessible protein surfaces. In contrast, P_0_, despite its large volume (2445.4 Å^3^), was positioned at the α/β interface and is likely inaccessible under dimerized conditions. This integrative analysis, which combines geometric and physicochemical features, provided a rational basis for screening structurally viable pockets for subsequent CPPF docking simulations. The spatial distribution of the identified binding pockets is visualized in Fig 1D. For the α/β-tubulin dimer, Protenix identified five candidate poses, with pose 1 selected as having the most favorable interaction geometry (Table 1). In this pose, CPPF inserts into a cleft between the α- and β-subunits, with its pyridinyl nitrogen forming a polar contact with VAL236 on β-tubulin at a distance of 2.9 Å (Fig 2A, bottom). This interaction was corroborated by PLIP analysis, which identified a hydrogen bond between the same CPPF atom and VAL236 across multiple poses (distance range: 2.05–2.95 Å), reinforcing the plausibility of this anchoring site (S2 Table).

### Structure prediction and validation

In α-tubulin-only models, CPPF binding was more variable across platforms. Protenix placed the ligand near ASN257, forming a hydrogen bond at ~3.0 Å (Fig 2B), while RFAA predicted an adjacent but more surface-exposed binding mode (Fig 2C) with a hydrogen bond to ALA270 and halogen bonds to SER241 (S2 Table). These differences may reflect the lower structural constraints in the monomeric α-tubulin models. Conversely, β-tubulin predictions were more consistent across tools. In both Protenix and RFAA models, CPPF occupied a similar hydrophobic pocket involving residues TYR200, GLU198, LEU253, and VAL236, forming recurrent hydrophobic and hydrogen bonding interactions (Fig 2D–2E). In addition, Umol independently predicted a comparable CPPF binding mode on the β-tubulin monomer (S5A Fig), centering around residue VAL236 also recurrent in Protenix and RFAA results, thereby reinforcing the consistency and robustness of this predicted anchoring site across platforms. Taken together, these consistent interaction profiles across Protenix, RFAA, and Umol strengthen the confidence in the β-tubulin pocket as a stable and conserved binding site for CPPF.

To assess the reproducibility and convergence of predicted CPPF binding poses across oligomeric states, we compared five Protenix-generated models for the α/β-tubulin dimer, α-tubulin monomer, and β-tubulin monomer. Monomeric predictions consistently yielded lower RMSD values (0.578–1.028Å) than dimeric counterparts (1.318–1.950Å), as shown in Table 1 and S5B–S5D Figs. For example, pose 4 achieved the best alignment in the β-tubulin monomer (0.578Å), while the corresponding dimer model showed 1.869Å. Averaged across all poses, β-tubulin models exhibited the highest structural convergence (mean RMSD = 0.682Å), followed by α-tubulin (0.949Å), and dimers (1.766Å).

Confidence metrics (pTM and ipTM) were high across all monomeric models, with α-tubulin showing slightly higher average scores (pTM = 0.9726, ipTM = 0.9835) than β-tubulin (pTM = 0.9696, ipTM = 0.9814). This may reflect the more regular backbone architecture of α-tubulin, as AI predictors tend to assign higher confidence to structurally conserved domains. Nevertheless, the tighter fit and lower RMSD values observed in β-tubulin models suggest a more constrained and reproducible binding pocket geometry for CPPF.

When focusing specifically on monomeric subunits, β-tubulin consistently outperformed α-tubulin in pose reproducibility. RMSD values for β-tubulin ranged from 0.578 to 0.841 Å, compared to 0.872 to 1.028 Å for α-tubulin. Pose 4 was optimal for β-tubulin (0.578 Å), while pose 2 yielded the lowest RMSD for α-tubulin (0.872 Å). These findings indicate that although α-tubulin predictions were marginally more confident overall, β3-tubulin provided a more consistent spatial environment for CPPF docking across replicate models.

To further evaluate pose-level agreement, cross-platform structural overlays were generated. As illustrated in S5 Fig, CPPF binding orientations were topologically conserved across different models, particularly in β-tubulin monomer and α/β-tubulin dimer contexts. Coloring by pose ID (e.g., pale green for pose 0, purple for pose 1, etc.) revealed consistent pocket occupation, similar ligand orientations, and recurrent contact residues. These convergent features support the view that the β3-tubulin cleft represents a structurally consistent anchoring site for CPPF.

### Key residue contributions to ligand binding

**Fig 3:**
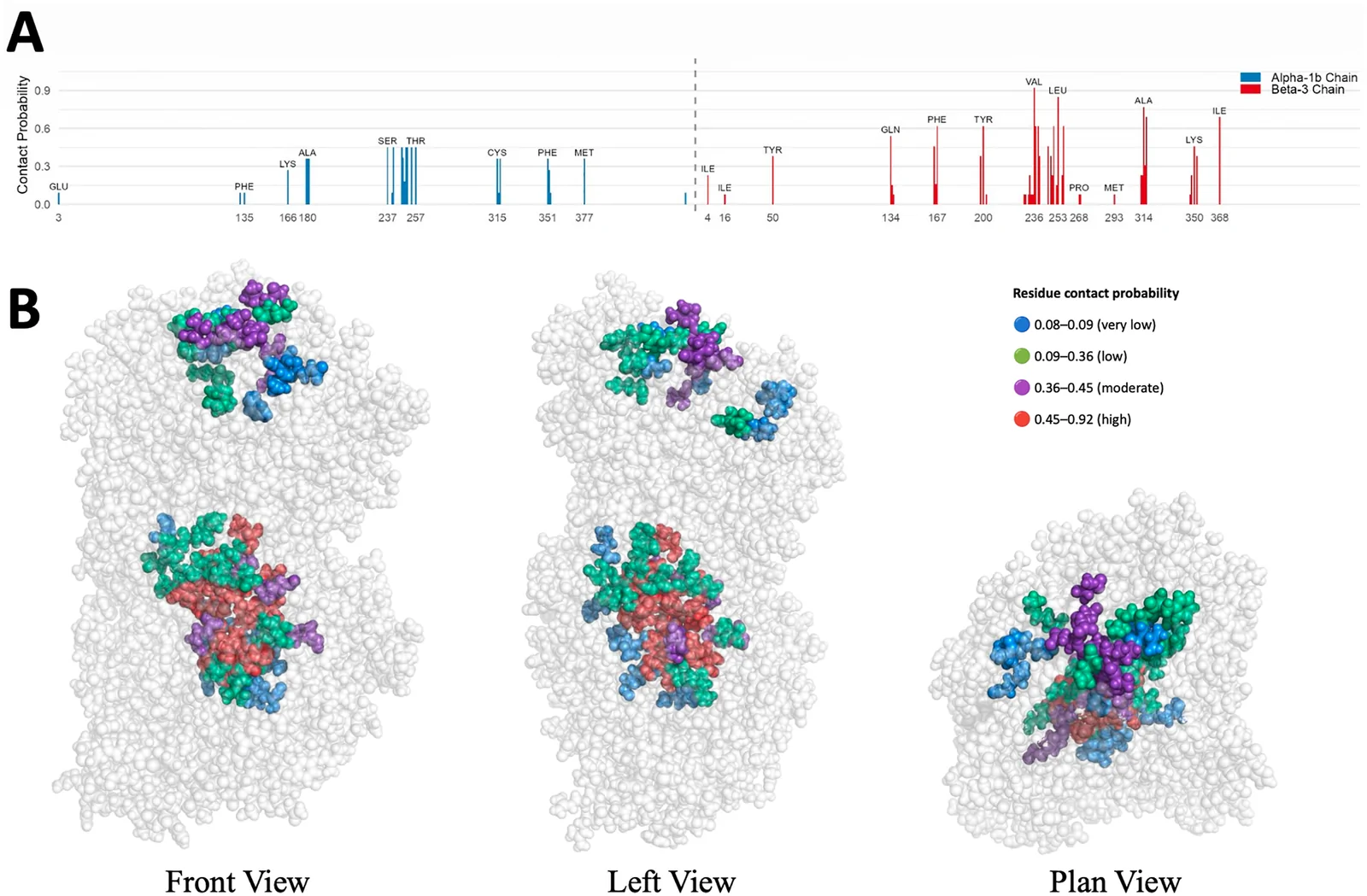
Multi-perspective analysis of predicted CPPF-binding residues on tubulin isoforms TUBA1B (α-tubulin) and TUBB3 (β-tubulin). All panels visualize predicted protein–ligand contacts derived from structural models using a 4 Å distance cutoff between heavy atoms of CPPF and tubulin residues. **(A)** Per-residue contact probability distribution along tubulin sequences. Bar plot summarizing the predicted contact probabilities of individual residues within TUBA1B (blue) and TUBB3 (red). Contact probabilities were computed across a panel of modeled α/β-tubulin–CPPF complexes. Peaks highlight residues with increased likelihood of interaction, with annotations indicating residue index and identity. The vertical dashed line separates α- and β-chain data. **(B)** 3D spatial distribution of predicted contact residues on the tubulin dimer. Three surface-rendered views (front, left, and plan) of the predicted TUBA1B–TUBB3 dimer, showing residues colored by contact probability bin: very low (0.08–0.09, green), low (0.09–0.36, blue), moderate (0.36–0.45, purple), and high (0.45–0.92, red). Only residues predicted to contact CPPF are colored; remaining protein regions are shown in transparent gray. This spatial mapping reveals clustering of interaction hotspots at the inter-chain interface.

In β-tubulin (TUBB3), PLIP consistently identified recurrent hydrophobic interactions involving VAL236, LEU253, ALA314, and VAL316, along with hydrogen bonds formed with polar residues such as TYR200, ASN256, and VAL236. Additionally, halogen bonding between CPPF and LEU135 was observed in select models, suggesting a role for CPPF’s halogen substituents in enhancing complex stability. In contrast, for α-tubulin (TUBA1B), PLIP detected hydrophobic interactions with residues including LEU241, LEU247, PHE254, and LEU317, and hydrogen bonds with ASN248 and ASN257 (S2 Table). However, these α-tubulin interactions appeared less frequently across predicted models (typically in 1–4 out of 10 predictions), and were more dispersed across surface-exposed helices and loops, indicating a more variable and less confined binding environment compared to β-tubulin. Mapping the contact probabilities onto the tubulin sequence revealed two primary clusters of CPPF-binding residues (Fig 3A). The first cluster, on α1B-tubulin (TUBA1B), included ALA180, SER237, and THR257, each with moderate contact probabilities of 0.36–0.45, appearing in approximately 4–5 out of 10 evaluated models. These residues are located on a solvent-accessible surface and were associated with fewer hydrophobic interactions in PLIP analysis, indicating a weaker and more variable binding mode.

In contrast, the second cluster on β3-tubulin (TUBB3) comprised VAL236, LEU253, and ALA314, which showed higher contact probabilities ranging from 0.75 to 0.92 and were present in ≥7 of the 10 models analyzed. These residues formed a contiguous patch at the α/β dimer interface. PLIP analysis supported their functional relevance by identifying consistent hydrophobic and hydrogen bonding interactions, reinforcing their role as anchoring hotspots for CPPF. This region is partially buried and topologically confined, supporting a stable binding configuration.

Three-dimensional projections (Fig 3B) revealed that high-contact β-tubulin residues clustered within an interfacial groove between α- and β-subunits, whereas α-tubulin contacts were more dispersed. Collectively, this multi-resolution analysis delineates a structurally and chemically coherent CPPF-binding interface enriched on β-tubulin, consistent across modeling platforms and interaction profiling.

### Structural comparison with the canonical colchicine binding site

To contextualize the predicted CPPF binding mode relative to the canonical colchicine site, we performed structural overlays between the experimentally determined colchicine-site crystal structure of α/β-tubulin (PDB: 4O2B) [39] and Protenix-predicted CPPF–tubulin models (S6 Fig). The overall structural alignment revealed that CPPF occupies a region proximal to the colchicine binding site at the α/β interface (S6A Fig). However, a close-up comparison of the binding pocket (S6B Fig) suggested that the predicted CPPF pose is positioned in a deeper β-tubulin cleft that is spatially distinct from the crystallographic colchicine-site-bound conformation. This geometric divergence was observed across both the heterodimer and monomer CPPF models.

### Interface stability and energetic evaluation

PDBePISA analysis of RFAA-predicted CPPF–tubulin complexes revealed compact but energetically favorable binding interfaces. In the CPPF–TUBB3 complex, 16 protein atoms (0.5%) and 11 residues (2.4%) contributed to an interface area of 183.9 Å^2^, while all atoms of CPPF were engaged. The CPPF–TUBA1B complex showed a slightly smaller interface (171.2 Å^2^) with similar protein residue involvement. In both models, the ligand remained largely solvent-exposed (97.8% and 99.9% in TUBB3 and TUBA1B, respectively), suggesting peripheral binding rather than deep cavity insertion (Table 2).

**Table 2:** Interface characteristics of the CPPF–TUBB3 & CPPF–TUBA1B complex as identified by PDBePISA. PDBePISA analysis was conducted on ligand–protein complexes predicted by RFAA. The interface area, number of participating atoms and residues, solvent-accessible surface area, and Gibbs free energy (Δ G) were computed for both α- and β-subunits interacting with CPPF. Values in parentheses indicate the proportion relative to the total number of atoms or residues in the protein or ligand.

Parameter	CPPF–TUBB3		CPPF–TUBA1B	
	Protein (TUBB3)	Ligand (CPPF)	Protein (TUBA1B)	Ligand (CPPF)
Number of atoms (interface)	16 (0.5%)	4 (100%)	14 (0.4%)	4 (100%)
Number of residues (interface)	11 (2.4%)	1 (100%)	11 (2.5%)	1 (100%)
Solvent-accessible area [Å^2^]	49.8 (0.2%)	317.9 (97.8%)	13.5 (0.1%)	328.8 (99.9%)
Interface area [Å^2^]	183.9	—	171.2	—
Gibbs free energy [kcal/mol]	−0.4	—	−0.2	—

Thermodynamic analysis indicated weak but favorable interactions, with Gibbs free energy of dissociation (Δ G) estimated at –0.4 kcal/mol (TUBB3) and –0.2 kcal/mol (TUBA1B). Despite minimal protein surface burial, the consistent full engagement of the ligand in both complexes supports the presence of a structurally coherent but potentially transient interface.

At the residue level, PDBePISA identified LEU253 in TUBB3 as a key contact residue, forming a hydrophobic interaction with CPPF at 1.39 Å. In the TUBA1B complex, multiple residues including THR239 and ILE238 were involved, with contact distances ranging from 1.10 to 1.81 Å. These contacts predominantly involved nonpolar atoms and lacked evidence of hydrogen bonds or salt bridges, indicating a hydrophobic interface without significant polar stabilization.

### Molecular dynamics and binding stability

**Fig 4:**
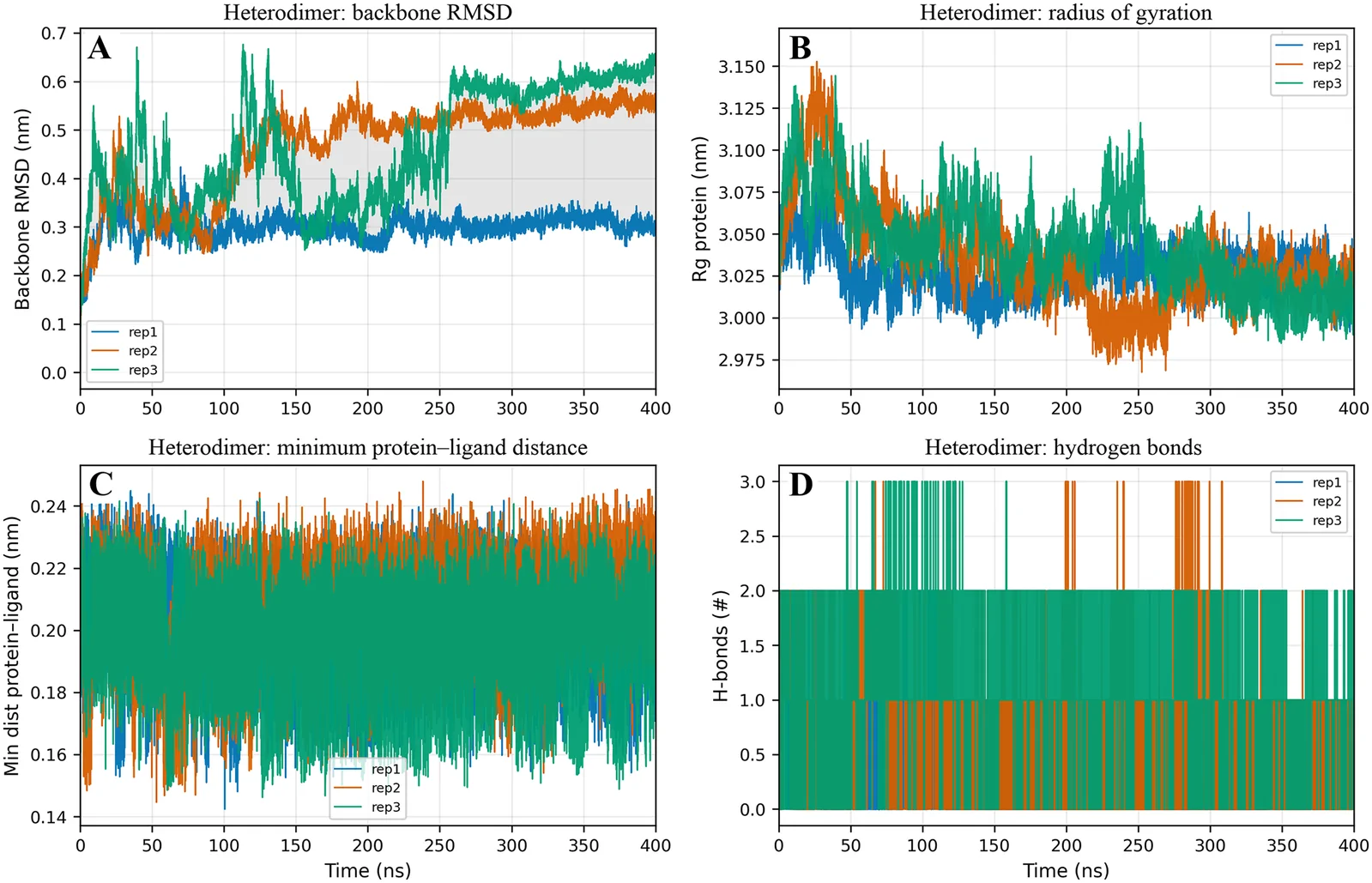
Molecular dynamics simulation of CPPF–α/β-tubulin heterodimer (400 ns; three replicates). Panels show: (A) backbone RMSD, (B) protein radius of gyration (Rg), (C) minimum protein–ligand distance, and (D) protein–ligand hydrogen bond count, each plotted as three replicate traces with an across-replicate min–max band.

**Fig 5:**
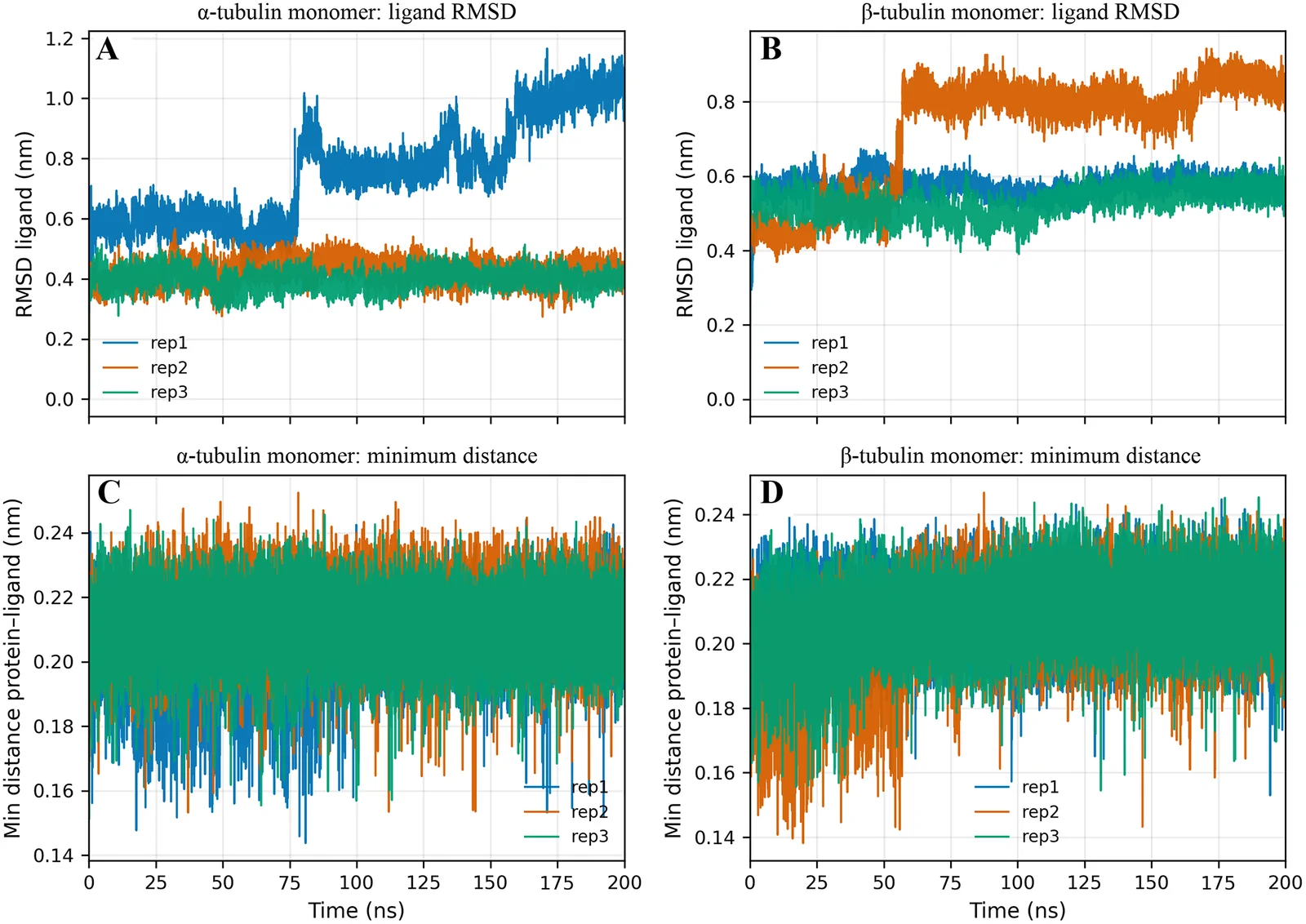
Monomer MD time series (200 ns; three replicates each). Panels show ligand RMSD for **(A)** α-tubulin and **(B)** β-tubulin monomers, and minimum protein–ligand distance for **(C)** α-tubulin and **(D)** β-tubulin monomers.

We next assessed binding stability using multi-replicate MD simulations, focusing on the assembled α/β heterodimer as the physiological binding context. Across three independent heterodimer replicates (400 ns each), CPPF maintained stable interfacial contact distances throughout the trajectories (Fig 4C), with minimum protein–ligand distances consistently in the 0.14–0.24 nm range. Backbone RMSD and Rg remained in physically reasonable ranges after periodic-boundary processing, indicating no spurious jump artefacts (Fig 4A–4B). Notably, the three replicates exhibited different convergence behaviors in backbone RMSD: replicate 1 reached a stable plateau (~0.3 nm) after approximately 50 ns, while replicates 2 and 3 showed continued conformational drift reaching ~0.55 and ~0.65 nm by 400 ns, suggesting ongoing structural exploration at this timescale. Importantly, the stable ligand contact distances across all three replicates indicate that the conformational variability primarily reflects backbone rearrangements rather than loss of the binding interface. Summary statistics for the final analysis windows are provided in S3 Table. To provide a complementary view of binding dynamics in monomeric contexts, we compared α- and β-tubulin monomer simulations (200 ns; three replicates each). Ligand RMSD traces revealed substantially greater variability and replicate-to-replicate spread in the α-tubulin monomer than in the β-tubulin monomer, while minimum distance traces indicated more consistent proximity in the β systems (Fig 5). Summary boxplots for the final 50 ns quantify these monomeric differences (S7 Fig). Notably, residue-level analysis during the final 50 ns (150–200 ns) indicated that CPPF did not consistently maintain proximity to the canonical β-tubulin pocket residues VAL236, LEU253, and ALA314 in isolated monomer simulations: mean minimum distances to LEU253 and ALA314 ranged from 0.64–1.36 nm and 0.90–1.59 nm across replicates, respectively, consistent with partial disengagement from the predicted binding site in the monomeric state. In the α-tubulin monomer, replicate 1 showed a marked ligand RMSD transition near ~80 ns, consistent with a ligand relocation event and underscoring the lower binding-site confinement in the monomeric context.

**Fig 6:**
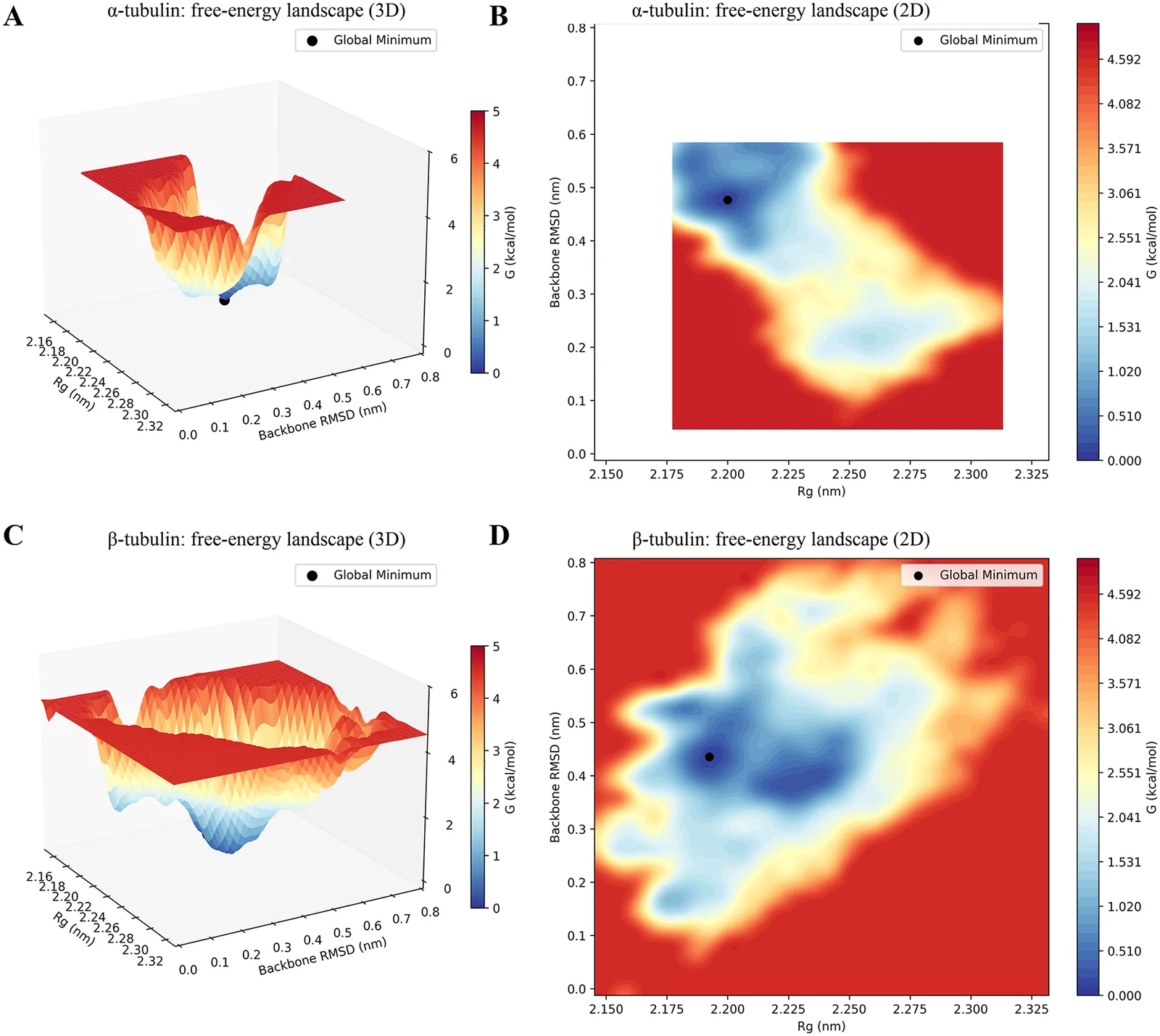
Free energy landscapes (FELs) of CPPF–tubulin simulations constructed using backbone RMSD and Rg as collective variables. The full 200 ns trajectories from all three replicates were concatenated within each class (α and β) to improve sampling. Panels show the α-tubulin landscape as (A) a 3D surface and (B) a 2D contour map, and the β-tubulin landscape as (C) a 3D surface and (D) a 2D contour map. α and β landscapes are plotted with shared axis limits and a shared free-energy colour scale (kcal/mol; capped at 5 kcal/mol) to enable direct visual comparison of basin depth and localization.

To further examine the conformational space sampled during simulation, we constructed two-dimensional free energy landscapes (FELs) using backbone RMSD and Rg as collective variables. The full 200 ns trajectories from all three replicates were concatenated within each class (α and β) prior to FEL construction to improve sampling coverage. Consistent with the time series analyses, the combined β landscape exhibited a deeper and broader low-energy basin than the combined α landscape (Fig 6), indicating a larger ensemble of energetically favorable conformations in the β binding context. The global minimum of the combined β landscape is located near Rg ≈ 2.19 nm and RMSD ≈ 0.43 nm, whereas the combined α landscape minimum occurs near Rg ≈ 2.20 nm and RMSD ≈ 0.48 nm, reflecting that the most probable α conformations remain more structurally drifted. Both landscapes are plotted with shared axis limits and a shared free-energy colour scale (kcal/mol; capped at 5 kcal/mol) to enable direct visual comparison.

Binding free energy was estimated using the MM-PBSA (GB) method applied to the heterodimer trajectories over the final 50 ns (350–400 ns, 51 frames at 1 ns intervals). Across the three replicates, the binding free energy components were: ΔVDW = −41.96 ± 1.33 kcal/mol, ΔEEL = −5.91 ± 2.22 kcal/mol, ΔEGB = +22.29 ± 0.63 kcal/mol, ΔESURF = −5.61 ± 0.23 kcal/mol, and ΔTOTAL = −31.19 ± 4.04 kcal/mol (mean ± SD across replicate averages). Bond energy terms that exhibited numerical overflow in individual sander outputs were flagged and excluded from per-frame decomposition; they do not affect ΔTOTAL because bond contributions are identical in the complex and receptor calculations and cancel upon subtraction. These values indicate a substantially more favorable binding interaction than the static PDBePISA estimate (ΔG = −0.4 kcal/mol), which reflects only the interface geometry of the predicted pose without accounting for conformational sampling or solvation dynamics.

### CPPF binding stability in the 6E7B (GTP-bound β-tubulin) conformational state

To examine whether CPPF binding is preserved across the major β-tubulin conformational states, we performed three independent 200 ns MD replicates on the 6E7B template, in which β-tubulin adopts the GTP-bound straight microtubule-lattice conformation (Methods and S8–S10 Figs). The protein–CPPF MD protocol was identical to that used for the 5IJ0 simulations to enable direct cross-system comparison.

Across all three 6E7B replicates, CPPF remained stably bound throughout the trajectories (S8 Fig). Over the final 50 ns analysis window, the backbone RMSD across replicates was 0.295±0.026 nm (per-replicate means: 0.270, 0.295, 0.321 nm), which is comparable to the lowest-drift 5IJ0 replicate (rep1, ~0.30 nm plateau) and substantially lower than 5IJ0 rep2/rep3 (~0.55 and ~0.65 nm). The tighter convergence is consistent with the more constrained backbone geometry of the straight lattice conformation relative to the curved soluble dimer. The minimum CPPF–protein distance was 0.203±0.014 nm across replicates (per-replicate means: 0.209, 0.214, 0.188 nm), within the 5IJ0 range of 0.14–0.24 nm, indicating that CPPF maintains continuous van-der-Waals contact with the binding pocket in the 6E7B conformation. The 2D free energy landscape constructed by Boltzmann inversion of the concatenated (RMSD, Rg) joint distribution exhibits a single well-defined global minimum at RMSD ≈ 0.27 nm, Rg ≈ 2.99 nm (S9 Fig). The larger Rg compared with the 5IJ0 β minimum (Rg ≈ 2.19 nm) reflects the extended geometry of the straight lattice conformation rather than loss of binding; basin localization is comparable to that of 5IJ0 β, indicating equivalent conformational confinement of the CPPF-bound state. MM-PBSA-GB analysis (gmx_MMPBSA, GB-OBC2, last 50 ns at ~1 ns sampling, identical protocol to the 5IJ0 simulations) yielded an across-replicate mean binding free energy of −27.82±5.44 kcal/mol (per-replicate: −21.80, −29.28, −32.38 kcal/mol), which is comparable to the 5IJ0 value of −31.19±4.04 kcal/mol, with overlapping 1σ intervals (S10 Fig).

Together, these supplementary findings indicate that CPPF accommodation is robust to the β-tubulin conformational state. The ligand maintains its position at the α/β-interface with high stability. Its backbone RMSD profile shows less drift than two of the three 5IJ0 replicates, while all other metrics remain comparable. This is consistent with the biochemical expectation that CPPF and the nucleotide cofactors occupy spatially distinct sites at the β-tubulin N-site and E-site, respectively, and with published experimental evidence that ligand binding at the overlapping colchicine site is not appreciably modulated by the β-tubulin nucleotide state.

## Discussion

This study models the predicted recognition site of α/β-tubulin dimer by CPPF through a structure-guided framework integrating AI-powered structure prediction (Protenix, RFAA, Umol), spatial contact profiling, atomic-resolution interaction annotation (PLIP), and molecular dynamics (MD) simulations. The analysis reveals that CPPF targets a composite α/β interfacial pocket dominated by β3-tubulin contacts, supported by comparative modeling across α/β-tubulin dimers and monomers (Figs 2 and S5 and S2 Table). The primary simulations employed the soluble, curved conformation of tubulin (PDB: 5IJ0), which represents the state relevant to microtubule-destabilizing ligands. The supplementary 6E7B simulations (3 × 200 ns) further indicate that CPPF binding is preserved in the GTP-bound straight microtubule-lattice conformation, with binding-pocket stability metrics comparable to the 5IJ0 replicates and a comparable MM-PBSA binding free energy (−27.82±5.44 vs −31.19±4.04 kcal/mol, overlapping 1σ intervals), supporting that CPPF accommodation is robust to the β-tubulin conformational state. Comparative modeling reveals that monomeric β3-tubulin exhibits superior structural convergence compared to α1B-tubulin and dimers. RMSD values for β3-tubulin range from 0.578 to 0.841 Å, lower than α1B-tubulin (0.872–1.028 Å) and dimers (1.318–1.950 Å) (Table 1), despite α-tubulin’s slightly higher pTM (0.9726) and ipTM (0.9835) scores versus β-tubulin’s (0.9696, 0.9814). PDBePISA analysis further supports this preference, indicating a larger interface area (183.9 Å^2^ vs. 171.2 Å^2^), greater atom engagement (0.5% vs. 0.4%), and a more favorable binding energy (ΔG = -0.4 vs. -0.2 kcal/mol) for β3-tubulin (Table 2), with tighter hydrophobic anchoring at LEU253 (1.39 Å) enhancing van der Waals complementarity. These findings indicate a geometrically optimized β3-tubulin site with higher structural consistency. Interaction profiling identifies a predicted β-tubulin binding pocket featuring VAL236, LEU253, and ALA314, with high contact probabilities (0.75–0.92) across models (Fig 3A), as supported by Protenix, RFAA, Umol, and PLIP analysis (S2 Table). These residues contribute to favorable hydrophobic and hydrogen-bond interactions (e.g., VAL316, TYR200, ASN256), including a halogen bond with LEU135 in select β3-tubulin complexes. In contrast, the α1B-tubulin region (ALA180, SER237, THR257) shows moderate contact probabilities (0.36–0.45) and occupies more solvent-exposed topographies with reduced polar interactions, suggesting a less stable binding environment. MD simulations further support that the assembled α/β heterodimer provides the relevant structural framework for stable CPPF accommodation. In isolated β-tubulin monomer simulations (200 ns), CPPF did not consistently maintain proximity to the canonical pocket residues over the trajectory. During the final 50 ns, the mean minimum distances to LEU253 and ALA314 ranged from 0.64–1.36 nm and 0.90–1.59 nm across replicates, respectively, indicating partial disengagement from the predicted binding site in the monomeric state. In contrast, within the α/β-tubulin heterodimer (400 ns; three independent replicates), CPPF maintained stable interfacial contacts throughout the simulations with consistent minimum protein–ligand distances of 0.14–0.24 nm (Fig 4), and a mean MM-PBSA binding free energy of −31.19±4.04 kcal/mol. The deeper and broader free energy basin observed for β-tubulin in the FEL analysis further supports that heterodimer formation and the resulting interfacial geometry are necessary for stable CPPF accommodation (Fig 6). These findings suggest that the biological context of the assembled dimer, rather than the isolated subunit, is the relevant structural framework for CPPF binding.

Prior MM-GBSA studies of colchicine-site ligands bound to human αβ-tubulin heterodimers report endpoint binding free energies on the order of tens of kilocalories per mole under implicit-solvent end-state protocols (for example, −51.95 to −64.45 kcal/mol for DAMA-colchicine across tubulin isotypes [40]). Absolute magnitudes are not directly comparable across studies, as they depend on the choice of ligand, force field, implicit solvent model (GB versus PB), trajectory length, and entropy approximation. Our ensemble-averaged MM-PBSA ΔTOTAL for CPPF (−31.19±4.04 kcal/mol) is of the same order of magnitude as, though quantitatively lower than, these DAMA-colchicine benchmarks [40]; the offset is consistent with expected differences in ligand chemistry (CPPF is smaller and less buried than DAMA-colchicine) and protocol details, and this comparison is intended to establish scale rather than direct affinity ranking. Our result nevertheless remains substantially more favorable than the PDBePISA single-structure interface estimate that omits conformational sampling [28]. Together with docking evidence placing CPPF at the colchicine-site region [20], these comparisons contextualize the present energetics relative to prior in silico studies of colchicine-class ligands.

The stable interaction between the β3-tubulin and CPPF is consistent with Han et al.’s (2020) prediction that CPPF binds at the colchicine site [20], where it can depolymerize microtubules and suppress growth in multidrug-resistant cancer cell lines, suggesting a mechanism distinct from paclitaxel’s microtubule stabilization [22]. This β-tubulin-dominant contact pattern may help explain CPPF activity in multidrug-resistant cellular contexts, including TUBB3-enriched tumors such as glioblastoma models, where TUBB3 overexpression is associated with chemoresistance [8]. The PDBePISA static estimate (ΔG = -0.4 kcal/mol) reflects only the interface geometry of a single predicted pose; MM-PBSA calculations incorporating conformational sampling yield a substantially more favorable ΔTOTAL of -31.19 ± 4.04 kcal/mol, supporting a stable binding interaction that warrants further experimental validation. Notably, the predicted CPPF binding pocket diverges from vinblastine’s lateral interface engagement, suggesting a unique tubulin interaction mode that may mitigate resistance mechanisms associated with traditional microtubule inhibitors [20]. Given that CPPF occupies a β-tubulin cleft partially overlapping with the colchicine site and exhibits potential stabilizing contacts at the α/β dimer interface (inferred from PDBePISA interface area, Table 2), it is plausible that such binding may interfere with protofilament assembly or destabilize lateral contacts critical for microtubule polymerization. This contrasts with paclitaxel, which binds along the microtubule lumen. These distinctions raise the possibility that CPPF may reverse paclitaxel resistance or enhance synergistic effects in combination therapies. However, functional assays, such as microtubule polymerization kinetics, cell-based synergy tests, or MDR reversal studies, are required to validate these hypotheses. The structural overlay with the crystallized colchicine-site crystal structure of α/β-tubulin (PDB: 4O2B) [39] further refines this interpretation (S6 Fig). Although CPPF is positioned near the broader colchicine-site region at the α/β interface, the Protenix-predicted CPPF pose occupies a deeper β-tubulin cleft that is spatially distinct from the crystallographic colchicine-site-bound conformation. This divergence may reconcile an apparent discrepancy in the literature: although prior AutoDock modeling suggested partial overlap between CPPF and colchicine binding sites [20], CPPF remains effective in colchicine-resistant cancer cell contexts [20]. The distinct binding geometry identified here suggests that CPPF may engage β-tubulin residues outside the canonical colchicine-bound pose, potentially helping to circumvent colchicine-specific resistance mechanisms. This interpretation is consistent with the observed MDR-bypassing activity of CPPF and supports a binding mode related to, but functionally distinct from, classical colchicine-site inhibitors.

The multi-conformational compatibility of the CPPF binding pocket has a further mechanistic implication. Whereas paclitaxel binds a lateral inter-protofilament interface that is only fully formed once tubulin has polymerized into the microtubule lattice [11,12], and colchicine-site occupancy is coupled to the curved-to-straight transition of the α/β-heterodimer [16,17], the interfacial site occupied by CPPF is present in both the soluble curved dimer (5IJ0) and the straight lattice-related conformation (6E7B). CPPF retains stable binding in both conformational states across the four independent stability metrics reported here: backbone RMSD, minimum protein–ligand distance, FEL basin localization, and MM-PBSA binding free energy (−31.19±4.04 vs −27.82±5.44 kcal/mol). This cross-state accessibility supports the hypothesis that CPPF may engage tubulin at multiple stages of microtubule dynamics, potentially both antagonizing curved-to-straight polymerization from soluble dimers and destabilizing already-assembled lattice; definitive mechanistic distinction from stage-restricted binders would require dynamic assembly assays or single-molecule imaging.

While these analyses provide useful structural insights, limitations include the finite MD timescale (200 ns monomers; 400 ns heterodimers; 200 ns 6E7B supplementary replicates) and the intrinsic uncertainty of AI-based structure prediction and docking in capturing long-timescale conformational transitions [41,42]. The observed replicate-to-replicate variability in backbone drift in the heterodimer trajectories further indicates ongoing structural exploration at the 400 ns timescale, even when the binding interface remains stable. These discrepancies necessitate experimental validation. Although the supplementary 6E7B simulations indicate that CPPF binding is preserved in the GTP-bound straight β-tubulin conformation, a fully explicit GMPCPP-parameterized comparison, which would resolve any direct chemical contribution of the bound nucleotide at the E-site, remains a refinement for follow-up work. In summary, this study presents computational evidence that CPPF engages a composite α/β interface pocket characterized by primary contacts with β3-tubulin. The enhanced structural convergence, favorable energetics, and compact binding interface of β3-tubulin suggest that it contributes substantially to CPPF recognition and its potential microtubule-depolymerizing and MDR-bypassing effects. These computational predictions provide useful reference points for future analog design and warrant further validation through experimental assays.

## Supporting information

S1 Fig Toxicity and off-target activity profile of CPPF predicted by Pro-Tox 3.0. Radar plot showing the predicted toxicity probabilities of CPPF across a panel of toxicity endpoints and off-target targets. The blue line with filled area represents the toxicity probabilities of CPPF, while the orange line with filled area shows the average activity probabilities of known molecules from the ProTox 3.0 training dataset.
(TIF)

S2 FigStructural alignment and benchmark validation of Protenix-predicted β-tubulin–nocodazole complexes relative to the experimental crystal structure (PDB: 5CA1).Structural alignment was performed between the experimentally determined crystal structure of nocodazole-bound β-tubulin (PDB: 5CA1) and corresponding computational models predicted by Protenix. (A) Overview of the experimental β-tubulin–nocodazole structure overlaid with Protenix-predicted complexes (poses 0–2). (B) Close-up view of the ligand–receptor binding interface comparing the experimental structure and predicted poses. The crystal structure of β-tubulin in complex with nocodazole was derived from the T2R-TTL-nocodazole assembly (PDB: 5CA1). Cofactors and non-target protein components were omitted in PyMOL to enhance visual clarity. Structural alignments were conducted independently between PDB 5CA1 and each of the Protenix-predicted poses 0, 1, and 2. Color coding: experimental β-tubulin–nocodazole, magenta; predicted pose 0, cyan; predicted pose 1, blue; predicted pose 2, purple.(TIF)

S3 FigStructural alignment of predicted CPPF–tubulin complexes with experimental PDB structures.Predicted CPPF–tubulin complexes were structurally aligned to experimentally determined tubulin structures from the Protein Data Bank (PDB). (A) Alignment of the α/β-tubulin dimer (PDB ID: 5IJ0) with the CPPF–α/β-tubulin dimer predicted by Protenix. (B) Alignment of α-tubulin (TUBA1B) from the PDB with CPPF-bound α-tubulin predicted by Protenix. (C) Alignment of α-tubulin predicted by RFAA with the experimental structure. (D) Overlay of the experimental α-tubulin structure with Protenix- and RFAA-predicted CPPF-bound models. (E) Alignment of β-tubulin (TUBB3) from the PDB with the CPPF–β-tubulin complex predicted by Protenix. (F) Alignment of β-tubulin predicted by RFAA with the experimental structure. (G) Overlay of the experimental β-tubulin structure with Protenix- and RFAA-predicted CPPF-bound models. For all alignments, the best representative Protenix pose (pose 1) was selected. Color scheme: PDB structures (pink for α-tubulin, blue for β-tubulin), Protenix predictions (purple), and RFAA predictions (yellow). RMSD values are reported in Table 1. All structural alignments and visualizations were performed using PyMOL.(TIF)

S4 FigStructural comparison of the Protenix-predicted CPPF–6E7B complex with the 5IJ0-predicted complex.(A) Overall view of the Protenix-predicted CPPF–α/β-tubulin complex generated from the 6E7B (β-GMPCPP, straight microtubule-lattice) template, used as the MD starting structure for the supplementary 6E7B simulations (Methods and S8–S10 Figs). (B) Zoomed-in view of the predicted CPPF-binding pocket in the 6E7B-derived complex, showing CPPF (yellow) adjacent to β-tubulin VAL236 (orange; closest heavy-atom distance 2.48 Å) and LEU253 (teal; closest heavy-atom distance 2.80 Å), the same two residues identified as principal CPPF contacts in the 5IJ0 analysis (Fig 2A and S2 Table). (C) Structural overlay of the 6E7B-predicted complex (blue β-tubulin backbone; yellow CPPF; orange VAL236/LEU253 side chains) with the 5IJ0-predicted complex (salmon β-tubulin backbone; magenta CPPF; purple VAL236/LEU253 side chains), aligned on the β-tubulin backbone Cα atoms (PyMOL align; RMSD = 0.212 Å over 382 atom pairs). The close superposition of both the protein backbone and the two independently predicted CPPF poses indicates that the predicted binding site is conserved between the two β-tubulin conformational states, independent of and complementary to the MD/MM-PBSA-based evidence in S8–S10 Figs. Cofactors and non-target chains hidden for visual clarity.(TIF)

S5 FigPredicted CPPF binding poses on tubulin generated by Umol and Protenix.Predicted binding orientations of CPPF on tubulin were generated using Umol and Protenix. (A) Binding pose of CPPF on β-tubulin predicted by Umol. (B–D) Five predicted poses (pose 0–4) of CPPF in complex with the α/β-tubulin dimer (B), α-tubulin monomer (C), and β-tubulin monomer (D) generated by Protenix, aligned to their respective experimental PDB structures. Structures were color-coded as follows: PDB α-tubulin (pink), PDB β-tubulin (blue), Protenix pose 0 (pale green), pose 1 (purple), pose 2 (lavender blue), pose 3 (wheat), and pose 4 (gray). Root-mean-square deviation (RMSD) values for each pose are reported in Table 1. All structural visualizations and alignments were performed using PyMOL.(TIF)

S6 FigStructural comparison of the crystallized colchicine-site crystal structure of α/β-tubulin (PDB: 4O2B) with Protenix-predicted CPPF-bound β-tubulin models.(A) Overall view of the experimental colchicine-site crystal structure of α/β-tubulin overlaid with Protenix-predicted CPPF-bound complexes, including the full α/β-tubulin heterodimer and the isolated β-tubulin monomer. (B) Zoomed-in view of the ligand-binding pocket, comparing the experimental colchicine-site-bound complex with the predicted CPPF–β-tubulin interface from both the heterodimer and monomer models. The colchicine-site crystal structure of α/β-tubulin was obtained from the Protein Data Bank (PDB: 4O2B) [39]. Cofactors and non-target protein components were hidden in PyMOL to enhance visual clarity. Structural alignments were performed between PDB 4O2B and the Protenix-predicted CPPF-bound α/β-tubulin heterodimer and β-tubulin monomer models. Color scheme: experimental α/β-tubulin–colchicine-site-bound complex, pink; Protenix-predicted α/β-tubulin heterodimer–CPPF complex, yellow; Protenix-predicted β-tubulin monomer–CPPF complex, cyan.(TIF)

S7 FigMonomer MD summary metrics over the final 50 ns (boxplots).Boxplots comparing α- and β-tubulin monomer simulations (200 ns; three independent replicates each) over the final 50 ns window (150–200 ns). (A) Minimum protein–ligand distance. (B) Mean binding-site RMSF (nm) averaged over residues VAL236, LEU253, and ALA314. (C) Ligand RMSD. (D) Protein radius of gyration (Rg). For each metric, distributions pool all frames within the window per replicate; boxplots summarize α versus β classes (three replicate values per class for panel B; pooled frame distributions for panels A, C, and D). These summaries complement the full-trajectory time series in the main text (Fig 5) and support greater binding-site variability and weaker late-stage confinement in α-tubulin monomer simulations relative to β-tubulin.(TIF)

S8 FigCPPF binding stability in the 6E7B (β-GTP/microtubule-lattice straight) conformation — MD time series.Three independent 200 ns MD replicates of the CPPF–αβ-tubulin complex starting from a Protenix-predicted pose aligned to PDB 6E7B (Methods). (A) Backbone RMSD; (B) protein radius of gyration; (C) minimum CPPF–protein distance; (D) CPPF–protein hydrogen-bond count. Per-replicate traces: rep1 blue, rep2 orange, rep3 green; gray shaded band: across-replicate min–max envelope. The MD protocol mirrors the 5IJ0 simulations (AMBER99SB-ILDN + GAFF2/RESP2; TIP3P water; 0.15 M NaCl; 2 fs timestep; cofactors not retained in the production topology). Across all three replicates, last-50-ns mean values are: backbone RMSD = 0.295±0.026 nm, minimum CPPF–protein distance = 0.203±0.014 nm. Corresponding window statistics are provided in S4 Table.(TIF)

S9 Fig6E7B two-dimensional free energy landscape (FEL).2D FEL constructed by Boltzmann inversion of the concatenated (Rg, backbone RMSD) joint distribution from all three 6E7B replicates. Energies are plotted in kcal/mol and capped at 5 kcal/mol to match the visualization convention of Fig 6 and S11 Fig. Global minimum (black dot): Rg ≈ 2.99 nm, backbone RMSD ≈ 0.27 nm. The higher Rg relative to the 5IJ0 β minimum (Rg ≈ 2.19 nm) reflects the extended geometry of the straight lattice conformation rather than loss of binding; basin localization is comparable to that of 5IJ0 β in Fig 6, indicating equivalent conformational confinement of the CPPF-bound state.(TIF)

S10 FigMM-PBSA-GB binding free energy comparison between 5IJ0 and 6E7B.Bar comparison of CPPF binding free energy ($\Delta G_{MM-PBSA-GB}$) between the 5IJ0 heterodimer simulations (3 × 400 ns; blue) and the 6E7B simulations (3 × 200 ns; orange). Bar height: across-replicate mean. Error bars: across-replicate s.d. Black dots: per-replicate ΔG values. Both systems were analyzed with the identical MM-PBSA-GB protocol (gmx_MMPBSA v1.5 + ; GB-OBC2 with igb = 5, intdiel = 1.0, extdiel = 78.5; ff99SB + GAFF; last 50 ns at ~1 ns sampling, ~50 snapshots per replicate). Across-replicate means: 5IJ0 = −31.19±4.04 kcal/mol; 6E7B = −27.82±5.44 kcal/mol. The two 1σ intervals overlap completely, indicating comparable CPPF binding energetics across the two β-tubulin conformational states.(TIF)

S11 FigSupplementary free energy landscapes (Rg–backbone RMSD) for monomer simulations.Two-dimensional free energy landscapes (FELs) for CPPF–tubulin monomer simulations constructed using backbone RMSD and Rg as collective variables. The full 200 ns trajectories from all three replicates were concatenated within each monomer class prior to SHAM analysis. Landscapes are plotted in kcal/mol with a colour scale capped at 5 kcal/mol (same convention as the 5IJ0 FEL comparison in Fig 6). (A) Combined α-tubulin monomer landscape. (B) Combined β-tubulin monomer landscape. The β-tubulin landscape exhibits a deeper and broader low-energy basin than the α-tubulin landscape, consistent with Fig 6.(TIF)

S1 TableProteinsPlus-predicted pocket properties for the α/β-tubulin heterodimer.This table reports the complete raw quantitative output from ProteinsPlus for all 29 surface pockets (P_0_–P_28_) detected on the α/β-tubulin dimer (PDB ID: 5IJ0). For each pocket, geometric and physicochemical descriptors are provided, including pocket volume, surface area, depth, surface-to-volume ratio, SimpleScore, DrugScore, hydrogen-bond acceptor/donor counts, hydrophobic interaction counts, metal-ion involvement, and the relative composition of nonpolar/polar/apolar surface areas. Nine pockets (P_0_–P_8_) with DrugScore > 0.7 were considered potentially druggable and were prioritized for downstream inspection. The spatial localization of these pockets on the tubulin structure is visualized in Fig 1D.(PDF)

S2 TableResidue-level interaction summary between CPPF and α/β-tubulin across predicted models identified by PLIP.This table summarizes the predicted non-covalent interactions between the small molecule CPPF and α- and β-tubulin residues as identified using the Protein–Ligand Interaction Profiler (PLIP). Multiple binding poses were analyzed across different structural models, including Protenix-based docking predictions and RFAA-predicted tubulin structures. For each interaction, the interaction type (hydrophobic interaction, hydrogen bond, or halogen bond), interacting residue (with chain identifier and residue number), and associated geometric descriptors (interatomic distance and interaction vector angles) are reported.(PDF)

S3 TableSummary statistics for MD simulation observables over the final analysis window.Per-system summary of key MD-derived metrics computed over the final 50 ns window (350–400 ns for heterodimer replicates; 150–200 ns for monomer replicates). Metrics include backbone RMSD, ligand RMSD, minimum protein–ligand distance (mindist_pl), hydrogen bond count (hbond_num), radius of gyration (Rg), solvent-accessible surface area (SASA), and per-residue RMSF. Values represent mean ± SD across frames within the specified window. All distances are in nm and SASA in nm^2^.(CSV)

S4 TablePer-replicate window statistics for the 6E7B MD simulations.Per-replicate summary of key MD-derived metrics computed over the final 50 ns analysis window (150–200 ns) of the three 6E7B simulations. Metrics reported for each replicate include backbone RMSD, minimum CPPF–protein distance (mindist_pl), hydrogen bond count (hbond_num), radius of gyration (Rg), and solvent-accessible surface area (SASA). Values represent mean ± SD across frames within the analysis window. All distances are in nm and SASA in nm^2^.(CSV)
