# Supplementary material for: Integrative AI-assisted modeling suggests CPPF binding at a composite α/β-tubulin interface pocket dominated by β-tubulin contacts
Source: PLoS Comput Biol. 2026 Sep 18;22(9):e1014804. doi: 10.1371/journal.pcbi.1014804 (PMC13607999; doi:10.1371/journal.pcbi.1014804)
Supplement: Supplementary file 1 [file pcbi.1014804.s012.pdf]

S1 Table. **Structural mapping of the binding pockets onto the α/β-tubulin dimer structure.**

This table reports the complete quantitative output for all 29 surface pockets (P_0–P_28) detected on the α/β-tubulin dimer (PDB ID: 5IJ0) by ProteinsPlus. Geometric and physicochemical descriptors include volume, surface area, depth, surface-to-volume ratio, SimpleScore, DrugScore, hydrogen-bond acceptor/donor counts, hydrophobic interactions, metal-ion involvement, and relative surface composition. Nine pockets (P_0–P_8) with DrugScore values >0.7 were prioritized for downstream inspection; P_2 had the highest DrugScore (0.90). The spatial distribution of these nine selected pockets is shown in Fig 1D.

Pocket	Volume (Å³)	Surface (Å²)	Depth (Å)	Surf/Vol	SimpleScore	DrugScore	Acceptors	Donors	Hydrophobic Interactions	Metal Ions	negAA (%)	posAA (%)	polarAA (%)	apolarAA (%)
P_0	2445.4	2396.02	40.86	0.979806984542406	0.56	0.808415	177	65	54	1	0.12	0.1	0.29	0.48
P_1	616.62	842.47	20.31	1.366270961045700	0.33	0.830405	65	18	22	0	0.11	0.06	0.36	0.47
P_2	559.2	771.2	36.79	1.3791130185980000	0.33	0.896846	43	21	29	0	0.05	0.08	0.32	0.55
P_3	553.24	776.88	18.36	1.4042368592292700	0.28	0.800914	38	23	14	0	0.07	0.03	0.41	0.48
P_4	437.9	582.11	16.84	1.32932176295958	0.23	0.742084	38	10	20	0	0.08	0.12	0.38	0.42
P_5	412.47	414.19	20.74	1.004170000242440	0.1	0.812516	53	9	2	0	0.07	0.05	0.34	0.54
P_6	407.7	601.47	17.54	1.4752759381898500	0.17	0.748267	42	17	17	0	0.15	0.04	0.38	0.42
P_7	333.39	330.03	23.92	0.9899217133087380	0.06	0.842805	40	10	5	0	0.03	0.06	0.38	0.53
P_8	303.39	331.08	19.65	1.0912686640957200	0.09	0.763716	21	5	9	0	0.0	0.04	0.43	0.52
P_9	233.75	280.36	14.02	1.1994010695187200	0.03	0.55773	24	4	11	0	0.2	0.15	0.2	0.45
P_10	219.35	261.2	14.27	1.190790973330290	0.06	0.565422	18	4	15	0	0.06	0.0	0.39	0.56
P_11	185.87	315.45	13.37	1.697153924786140	0.0	0.446528	16	13	11	0	0.11	0.06	0.67	0.17
P_12	179.71	214.14	11.24	1.191586444827780	0.0	0.422041	15	5	13	0	0.12	0.06	0.19	0.62
P_13	178.42	123.18	12.16	0.6903934536486940	0.0	0.43371	24	3	2	0	0.05	0.1	0.45	0.4
P_14	168.38	142.39	12.6	0.8456467513956530	0.0	0.459506	22	2	1	0	0.05	0.09	0.32	0.55
P_15	168.29	305.99	8.34	1.8182304355576700	0.0	0.289813	20	4	11	0	0.21	0.14	0.14	0.5
P_16	160.64	189.93	12.27	1.1823331673306800	0.0	0.427095	10	5	8	0	0.21	0.21	0.14	0.43
P_17	151.5	259.44	11.66	1.7124752475247500	0.0	0.402223	20	3	8	0	0.0	0.07	0.43	0.5
P_18	142.95	238.82	9.05	1.6706540748513500	0.0	0.294399	18	9	8	0	0.17	0.17	0.17	0.5
P_19	124.97	220.39	9.82	1.7635432503800900	0.0	0.320906	12	0	11	0	0.11	0.11	0.11	0.67
P_20	120.1	217.49	10.82	1.810907577019150	0.0	0.336709	12	5	6	0	0.09	0.0	0.45	0.45
P_21	114.54	221.5	10.08	1.933822245503750	0.0	0.296399	10	4	12	0	0.0	0.0	0.6	0.4
P_22	113.45	161.25	7.6	1.4213309828118100	0.0	0.214211	10	2	3	0	0.11	0.11	0.33	0.44
P_23	112.55	244.58	8.09	2.173078631719240	0.0	0.214779	14	8	8	0	0.11	0.22	0.33	0.33
P_24	106.4	195.04	7.65	1.833082706766920	0.0	0.202273	12	4	3	0	0.0	0.25	0.38	0.38
P_25	106.2	213.72	11.02	2.012429378531070	0.0	0.332298	11	5	2	0	0.18	0.18	0.18	0.45
P_26	103.42	147.51	7.01	1.4263198607619400	0.0	0.183956	14	5	7	0	0.1	0.0	0.5	0.4
P_27	102.82	237.26	6.71	2.307527718342740	0.0	0.16065	16	1	7	0	0.43	0.0	0.29	0.29
P_28	101.33	197.69	8.35	1.950952333958350	0.0	0.235549	8	1	12	0	0.2	0.2	0.1	0.5
