# Supplementary material for: Integrative AI-assisted modeling suggests CPPF binding at a composite α/β-tubulin interface pocket dominated by β-tubulin contacts
Source: PLoS Comput Biol. 2026 Sep 18;22(9):e1014804. doi: 10.1371/journal.pcbi.1014804 (PMC13607999; doi:10.1371/journal.pcbi.1014804)
Supplement: Supplementary file 2 [file pcbi.1014804.s013.pdf]

S2 Table. Residue-level interaction summary between CPPF and α/β-tubulin across predicted models using PLIP. This table lists the predicted non-covalent interactions between the small molecule CPPF and α/β-tubulin residues as identified by the PLIP. Multiple docking poses were generated from different structural models, including Protenix-based and RoseTTAFold-predicted tubulin structures. Each entry specifies the interaction type (hydrophobic interaction, hydrogen bond, or halogen bond), interacting residue (with chain and residue number), and the calculated spatial features such as distance and interaction vector angles.

Model	Interaction_Type	Residue	Resnr	ResChain	Distance	Angle
Protenix-Dimer-CPPF-Pose0	Hydrophobic Interactions	VAL181	181	A	3.93	-5.598, 2.671, 2.923
Protenix-Dimer-CPPF-Pose0	Hydrophobic Interactions	LEU253	253	B	3.77	-1.528, 9.311, -3.028
Protenix-Dimer-CPPF-Pose0	Hydrophobic Interactions	ASN256	256	B	3.63	-2.797, 3.831, -0.016
Protenix-Dimer-CPPF-Pose0	Hydrophobic Interactions	ALA314	314	B	3.89	-2.270, 9.850, -0.825
Protenix-Dimer-CPPF-Pose0	Hydrophobic Interactions	VAL316	316	B	3.27	-2.198, 11.161, -1.179
Protenix-Dimer-CPPF-Pose0	Hydrophobic Interactions	LYS350	350	B	3.59	-3.461, 2.396, 1.894
Protenix-Dimer-CPPF-Pose0	Hydrogen Bonds	VAL236	236	B	2.50	-1.453, 10.608, -3.378
Protenix-Dimer-CPPF-Pose1	Hydrophobic Interactions	GLN134	134	B	3.89	18.606, -1.393, -1.771
Protenix-Dimer-CPPF-Pose1	Hydrophobic Interactions	PHE167	167	B	3.42	19.900, -1.826, -1.703
Protenix-Dimer-CPPF-Pose1	Hydrophobic Interactions	TYR200	200	B	3.51	16.227, -1.582, -1.143
Protenix-Dimer-CPPF-Pose1	Hydrophobic Interactions	VAL236	236	B	3.08	17.950, -3.122, -0.265
Protenix-Dimer-CPPF-Pose1	Hydrophobic Interactions	THR237	237	B	3.46	17.950, -3.122, -0.265
Protenix-Dimer-CPPF-Pose1	Hydrophobic Interactions	LEU250	250	B	3.71	15.658, -0.862, -2.159
Protenix-Dimer-CPPF-Pose1	Hydrophobic Interactions	LEU253	253	B	3.68	9.447, -2.558, 3.679
Protenix-Dimer-CPPF-Pose1	Hydrophobic Interactions	ALA314	314	B	3.48	9.447, -2.558, 3.679
Protenix-Dimer-CPPF-Pose1	Hydrophobic Interactions	VAL316	316	B	3.91	10.017, -3.697, 3.116
Protenix-Dimer-CPPF-Pose1	Hydrogen Bonds	TYR200	200	B	2.37	12.057, -0.399, -0.003
Protenix-Dimer-CPPF-Pose1	Hydrogen Bonds	VAL236	236	B	2.05	12.761, -2.195, 1.207
Protenix-Dimer-CPPF-Pose2	Hydrophobic Interactions	VAL181	181	A	3.84	-2.921, -5.482, 3.766
Protenix-Dimer-CPPF-Pose2	Hydrophobic Interactions	LEU246	246	B	3.89	-2.937, -2.380, -0.436
Protenix-Dimer-CPPF-Pose2	Hydrophobic Interactions	ALA248	248	B	3.38	-8.904, -2.126, -3.478
Protenix-Dimer-CPPF-Pose2	Hydrophobic Interactions	LEU253	253	B	3.72	-8.904, -2.126, -3.478
Protenix-Dimer-CPPF-Pose2	Hydrophobic Interactions	ASN256	256	B	3.51	-3.753, -3.239, 0.273
Protenix-Dimer-CPPF-Pose2	Hydrophobic Interactions	VAL316	316	B	3.62	-10.989, -2.364, -1.797
Protenix-Dimer-CPPF-Pose2	Hydrophobic Interactions	LYS350	350	B	3.82	-3.753, -3.239, 0.273
Protenix-Dimer-CPPF-Pose2	Hydrophobic Interactions	ILE368	368	B	3.54	-10.989, -2.364, -1.797
Protenix-Dimer-CPPF-Pose2	Hydrogen Bonds	VAL236	236	B	2.92	-10.146, -2.022, -3.989
Protenix-Dimer-CPPF-Pose3	Hydrophobic Interactions	VAL181	181	A	3.84	-1.698, -2.784, -5.247
Protenix-Dimer-CPPF-Pose3	Hydrophobic Interactions	ALA248	248	B	3.99	-0.605, 6.833, -3.205
Protenix-Dimer-CPPF-Pose3	Hydrophobic Interactions	LEU253	253	B	3.91	-0.605, 6.833, -3.205
Protenix-Dimer-CPPF-Pose3	Hydrophobic Interactions	ASN256	256	B	3.67	-0.534, 0.426, -2.764
Protenix-Dimer-CPPF-Pose3	Hydrophobic Interactions	ALA314	314	B	3.95	-2.323, 5.989, -4.617
Protenix-Dimer-CPPF-Pose3	Hydrophobic Interactions	VAL316	316	B	3.35	-2.584, 7.267, -4.989
Protenix-Dimer-CPPF-Pose3	Hydrogen Bonds	VAL236	236	B	2.56	-0.865, 8.099, -3.570
Protenix-TUBA1B-CPPF-Pose0	Hydrophobic Interactions	LEU241	241	A	3.68	-9.959, -9.519, -0.852
Protenix-TUBA1B-CPPF-Pose0	Hydrophobic Interactions	LEU241	241	A	3.81	-8.532, -7.640, -1.028
Protenix-TUBA1B-CPPF-Pose0	Hydrophobic Interactions	LEU247	247	A	3.47	-9.286, -16.141, -7.998

Protenix-TUBA1B-CPPF-Pose0	Hydrophobic Interactions	LEU251	251	A	3.77	-8.532, -7.640, -1.028
Protenix-TUBA1B-CPPF-Pose0	Hydrophobic Interactions	PHE254	254	A	3.44	-9.408, -10.034, -2.004
Protenix-TUBA1B-CPPF-Pose0	Hydrophobic Interactions	LEU317	317	A	3.26	-7.692, -12.454, -6.325
Protenix-TUBA1B-CPPF-Pose0	Hydrophobic Interactions	LEU317	317	A	3.39	-6.789, -9.351, -4.619
Protenix-TUBA1B-CPPF-Pose0	Hydrophobic Interactions	LEU377	377	A	3.95	-6.789, -9.351, -4.619
Protenix-TUBA1B-CPPF-Pose0	Hydrogen Bonds	ASN257	257	A	3.04	-7.928, -17.123, -6.289
Protenix-TUBA1B-CPPF-Pose1	Hydrophobic Interactions	ILE237	237	A	3.42	2.076, 8.424, 5.921
Protenix-TUBA1B-CPPF-Pose1	Hydrophobic Interactions	LEU247	247	A	3.43	1.853, 12.029, 15.641
Protenix-TUBA1B-CPPF-Pose1	Hydrophobic Interactions	PHE254	254	A	3.13	1.006, 9.246, 8.028
Protenix-TUBA1B-CPPF-Pose1	Hydrophobic Interactions	LYS351	351	A	3.55	0.279, 11.184, 14.100
Protenix-TUBA1B-CPPF-Pose1	Hydrophobic Interactions	LYS351	351	A	3.51	0.548, 11.721, 15.337
Protenix-TUBA1B-CPPF-Pose1	Hydrogen Bonds	ASN248	248	A	3.38	-1.165, 10.400, 11.770
Protenix-TUBA1B-CPPF-Pose1	Hydrogen Bonds	ASN257	257	A	2.55	2.851, 11.816, 14.774
Protenix-TUBA1B-CPPF-Pose1	Hydrogen Bonds	ASN257	257	A	2.98	2.851, 11.816, 14.774
Protenix-TUBA1B-CPPF-Pose2	Hydrophobic Interactions	LEU166	166	A	3.94	1.044, -4.239, 7.559
Protenix-TUBA1B-CPPF-Pose2	Hydrophobic Interactions	ILE237	237	A	2.80	1.329, -5.424, 6.922
Protenix-TUBA1B-CPPF-Pose2	Hydrophobic Interactions	LEU241	241	A	3.50	1.945, -5.149, 9.572
Protenix-TUBA1B-CPPF-Pose2	Hydrophobic Interactions	LEU247	247	A	3.17	4.730, -15.389, 9.165
Protenix-TUBA1B-CPPF-Pose2	Hydrophobic Interactions	VAL249	249	A	3.56	2.816, -11.142, 7.503
Protenix-TUBA1B-CPPF-Pose2	Hydrophobic Interactions	LEU251	251	A	3.69	1.945, -5.149, 9.572
Protenix-TUBA1B-CPPF-Pose2	Hydrophobic Interactions	LYS351	351	A	3.48	4.730, -15.389, 9.165
Protenix-TUBA1B-CPPF-Pose2	Hydrophobic Interactions	LYS351	351	A	3.81	5.348, -13.246, 8.564
Protenix-TUBA1B-CPPF-Pose2	Hydrophobic Interactions	LEU377	377	A	3.19	2.778, -7.966, 5.708
Protenix-TUBB3-CPPF-Pose0	Hydrophobic Interactions	PHE167	167	B	3.25	1.904, -2.165, 5.517
Protenix-TUBB3-CPPF-Pose0	Hydrophobic Interactions	PHE167	167	B	3.69	2.987, -1.599, 6.130
Protenix-TUBB3-CPPF-Pose0	Hydrophobic Interactions	TYR200	200	B	3.35	1.855, -3.517, 5.316
Protenix-TUBB3-CPPF-Pose0	Hydrophobic Interactions	VAL236	236	B	3.25	1.855, -3.517, 5.316
Protenix-TUBB3-CPPF-Pose0	Hydrophobic Interactions	THR237	237	B	3.91	2.987, -1.599, 6.130
Protenix-TUBB3-CPPF-Pose0	Hydrophobic Interactions	LEU250	250	B	3.02	3.993, -3.753, 6.320
Protenix-TUBB3-CPPF-Pose0	Hydrophobic Interactions	LEU253	253	B	3.88	-0.457, -11.647, 9.112
Protenix-TUBB3-CPPF-Pose0	Hydrophobic Interactions	LEU253	253	B	3.16	0.231, -10.562, 8.643
Protenix-TUBB3-CPPF-Pose0	Hydrophobic Interactions	LEU253	253	B	3.72	3.299, -7.830, 4.825
Protenix-TUBB3-CPPF-Pose0	Hydrophobic Interactions	VAL316	316	B	3.80	-0.457, -11.647, 9.112
Protenix-TUBB3-CPPF-Pose0	Hydrophobic Interactions	ALA352	352	B	3.25	-0.457, -11.647, 9.112
Protenix-TUBB3-CPPF-Pose0	Hydrogen Bonds	VAL236	236	B	2.95	0.519, -8.859, 6.995
Protenix-TUBB3-CPPF-Pose1	Hydrophobic Interactions	GLN134	134	B	3.75	3.130, 0.164, -1.873
Protenix-TUBB3-CPPF-Pose1	Hydrophobic Interactions	PHE167	167	B	3.56	1.623, 0.862, -3.587
Protenix-TUBB3-CPPF-Pose1	Hydrophobic Interactions	MET233	233	B	3.60	1.971, 2.169, -3.367
Protenix-TUBB3-CPPF-Pose1	Hydrophobic Interactions	VAL236	236	B	3.48	6.326, 2.502, 0.417
Protenix-TUBB3-CPPF-Pose1	Hydrophobic Interactions	THR237	237	B	3.43	5.796, 2.074, -0.816
Protenix-TUBB3-CPPF-Pose1	Hydrophobic Interactions	LEU250	250	B	3.12	6.326, 2.502, 0.417
Protenix-TUBB3-CPPF-Pose1	Hydrophobic Interactions	LEU253	253	B	3.47	5.266, 6.177, 4.298
Protenix-TUBB3-CPPF-Pose1	Hydrophobic Interactions	LEU253	253	B	3.81	5.283, 6.788, 5.525
Protenix-TUBB3-CPPF-Pose1	Hydrophobic Interactions	ALA314	314	B	3.56	5.283, 6.788, 5.525

Protenix-TUBB3-CPPF-Pose1	Hydrophobic Interactions	ILE368	368	B	3.71	5.266, 6.177, 4.298
Protenix-TUBB3-CPPF-Pose1	Hydrogen Bonds	ASN165	165	B	3.32	5.458, 2.082, 3.558
Protenix-TUBB3-CPPF-Pose1	Hydrogen Bonds	TYR200	200	B	2.33	5.458, 2.082, 3.558
Protenix-TUBB3-CPPF-Pose1	Hydrogen Bonds	VAL236	236	B	3.30	5.025, 4.235, 2.980
Protenix-TUBB3-CPPF-Pose1	Halogen Bonds	LEU135	135	B	3.19	0.061, -4.277, -4.177
Protenix-TUBB3-CPPF-Pose2	Hydrophobic Interactions	PHE167	167	B	3.65	2.503, -1.329, -6.961
Protenix-TUBB3-CPPF-Pose2	Hydrophobic Interactions	TYR200	200	B	3.51	1.251, -1.162, -7.465
Protenix-TUBB3-CPPF-Pose2	Hydrophobic Interactions	VAL236	236	B	3.58	1.251, -1.162, -7.465
Protenix-TUBB3-CPPF-Pose2	Hydrophobic Interactions	LEU240	240	B	3.95	2.862, -0.201, -11.988
Protenix-TUBB3-CPPF-Pose2	Hydrophobic Interactions	LEU246	246	B	3.43	-2.674, -0.779, -15.440
Protenix-TUBB3-CPPF-Pose2	Hydrophobic Interactions	ALA248	248	B	3.84	2.194, -0.071, -13.214
Protenix-TUBB3-CPPF-Pose2	Hydrophobic Interactions	LEU250	250	B	3.26	3.406, -1.059, -9.154
Protenix-TUBB3-CPPF-Pose2	Hydrophobic Interactions	LEU253	253	B	3.61	1.058, -0.940, -8.815
Protenix-TUBB3-CPPF-Pose2	Hydrophobic Interactions	ALA352	352	B	3.65	-3.899, -0.860, -16.082
RoseTTAFold-TUBA1B-CPPF-Pose0	Hydrophobic Interactions	PHE169	169	A	3.69	3.891, -2.274, 0.622
RoseTTAFold-TUBA1B-CPPF-Pose0	Hydrophobic Interactions	PHE169	169	A	1.40	3.972, 2.196, -0.064
RoseTTAFold-TUBA1B-CPPF-Pose0	Hydrophobic Interactions	PHE169	169	A	2.20	4.011, 3.612, -0.167
RoseTTAFold-TUBA1B-CPPF-Pose0	Hydrophobic Interactions	PHE202	202	A	3.45	3.891, -2.274, 0.622
RoseTTAFold-TUBA1B-CPPF-Pose0	Hydrophobic Interactions	VAL204	204	A	1.91	3.648, -3.642, 0.653
RoseTTAFold-TUBA1B-CPPF-Pose0	Hydrophobic Interactions	VAL235	235	A	3.31	3.972, 2.196, -0.064
RoseTTAFold-TUBA1B-CPPF-Pose0	Hydrophobic Interactions	VAL235	235	A	3.29	4.011, 3.612, -0.167
RoseTTAFold-TUBA1B-CPPF-Pose0	Hydrophobic Interactions	ILE238	238	A	2.83	3.972, 2.196, -0.064
RoseTTAFold-TUBA1B-CPPF-Pose0	Hydrophobic Interactions	THR239	239	A	1.08	5.040, 7.719, -0.344
RoseTTAFold-TUBA1B-CPPF-Pose0	Hydrophobic Interactions	LEU242	242	A	3.78	6.271, 8.187, -0.409
RoseTTAFold-TUBA1B-CPPF-Pose0	Hydrogen Bonds	ALA270	270	A	3.35	5.600, -4.006, -0.633
RoseTTAFold-TUBA1B-CPPF-Pose0	Halogen Bonds	SER241	241	A	2.84	10.693, 10.002, -1.581
RoseTTAFold-TUBB3-CPPF-Pose0	Hydrophobic Interactions	VAL236	236	A	3.54	7.560, 6.655, 1.784
RoseTTAFold-TUBB3-CPPF-Pose0	Hydrophobic Interactions	THR237	237	A	3.81	4.230, 9.710, -0.383
RoseTTAFold-TUBB3-CPPF-Pose0	Hydrophobic Interactions	LEU240	240	A	2.92	4.230, 9.710, -0.383
RoseTTAFold-TUBB3-CPPF-Pose0	Hydrophobic Interactions	MET257	257	A	1.33	12.887, 1.809, -0.300
RoseTTAFold-TUBB3-CPPF-Pose0	Hydrophobic Interactions	PHE266	266	A	3.64	12.887, 1.809, -0.300
RoseTTAFold-TUBB3-CPPF-Pose0	Hydrophobic Interactions	VAL316	316	A	3.92	7.560, 6.655, 1.784
RoseTTAFold-TUBB3-CPPF-Pose0	Hydrophobic Interactions	ILE368	368	A	2.00	8.764, 5.806, 1.836
RoseTTAFold-TUBB3-CPPF-Pose0	Hydrophobic Interactions	ILE368	368	A	3.66	11.856, 2.689, -0.063
RoseTTAFold-TUBB3-CPPF-Pose0	Hydrogen Bonds	LEU253	253	A	2.56	14.469, 3.546, -0.195
